# Options for the Delivery of Intermittent Preventive Treatment for Malaria to Children: A Community Randomised Trial

**DOI:** 10.1371/journal.pone.0007256

**Published:** 2009-09-30

**Authors:** Margaret Kweku, Jayne Webster, Martin Adjuik, Samuel Abudey, Brian Greenwood, Daniel Chandramohan

**Affiliations:** 1 London School of Hygiene and Tropical Medicine, London, United Kingdom; 2 Navrongo Health Research Centre, Navrongo, Ghana; 3 Ghana Health Service, Hoehoe, Ghana; University of California Los Angeles, United States of America

## Abstract

**Background:**

Intermittent preventive treatment for malaria in children (IPTc) is a promising new intervention for the prevention of malaria but its delivery is a challenge. We have evaluated the coverage of IPTc that can be achieved by two different delivery systems in Ghana.

**Methods:**

IPTc was delivered by volunteers in six villages (community-based arm) and by health workers at health centres or at Expanded Programme on Immunisation outreach clinics (facility based) in another six communities. The villages were selected randomly and drugs were administered in May, June, September and October 2006. The first dose of a three-dose regimen of amodiaquine plus sulphadoxine-pyrimethamine was administered under supervision to 3–59 month-old children (n = 964) in the 12 study villages; doses for days 2 and 3 were given to parents/guardians to administer at home.

**Results:**

The proportion of children who received at least the first dose of 3 or more courses of IPTc was slightly higher in the community based arm (90.5% vs 86.6%; p = 0.059). Completion of the three dose regimen was high and similar with both delivery systems (91.6% and 91.7% respectively).

**Conclusion:**

Seasonal IPTc delivered through community-based or facility-based systems can achieve a high coverage rate with the support and supervision of the district health management team. However, in order to maximise the impact of IPTc, both delivery systems may be needed in some settings.

**Trial Registration:**

ClinicalTrials.gov NCT00119132

## Introduction

Seasonal intermittent preventive treatment for malaria in children (IPTc) is a promising new intervention for the prevention of malaria in children. Seasonal IPTc is safe and can reduce the burden of malaria substantially in countries of Sahelian West Africa. Dicko and colleagues in Mali showed that two treatments with suphadoxine-pyrimethamine (SP) given two months apart at the height of the malaria transmission season reduced the incidence of malaria by 40% in children 6 months to 10 years old [1]. Three treatment courses of SP plus a single dose of artesunate given during the high transmission season to children aged less than five years reduced the incidence of malaria by 86% in Senegal [2]. Three courses of SP given bimonthly reduced clinical malaria by 24% and anaemia (Hb<8.0 g/dl) by 30%, and six courses of artesunate plus amodiaquine (AQ) given monthly reduced clinical malaria by 69% and anaemia by 45% in the Hohoe district of Ghana [3].

A recent study in Senegal showed that IPTc using SP + AQ given for 3 days had the best protective efficacy compared to three other drug regimens. The adjusted hazard ratio for the incidence of malaria was 0.50 (95% CI 0.31,0.81) for SP (1 dose) +AQ given for 3 days, 0.90 (95% CI 0.60, 1.36) for SP (1 dose) + AS given for 3 days 1.13 (95% CI 0.76, 1.67) and AQ+AS given for 3 days when compared to SP +AS each given for one day [4].

Although IPTc with AQ and SP is safe and efficacious, there are concerns over how it can be delivered in an effective and sustainable way. In order to be effective, the delivery system must be accessible to the maximum number of target children, the SP/AQ must be offered to the correct children by the provider, and the correct regimen must be adhered to by the carer of the child. In terms of sustainability, the first consideration is whether it is possible to achieve effective delivery through routine public sector delivery systems. Delivery of IPTc during routine EPI/growth monitoring clinic visits must, therefore, be considered as one possible delivery system. However, there are two reasons to suspect that delivery of IPTc through this system may not reach the maximum target numbers in Ghana and other countries of West Africa. Firstly, the coverage of EPI vaccination is low in several countries of West Africa [5]. Secondly, in Ghana, immunisation and growth monitoring visits are more frequent amongst those under one year old than in those over one year (2004 Jasikan district annual report). It might be possible to increase the effectiveness of delivery through EPI/growth monitoring by using targeted health education campaigns to encourage caretakers to attend. If perceptions of IPTc are positive, then caretakers may be encouraged to use the clinics more regularly. Conversely, it is also plausible that the increased workload on the health services staff due to this integrated delivery of IPTc and growth monitoring could lead to programme fatigue.

Another option is a community based, volunteer delivery systems of the kind that has been used successfully for mass treatment of onchocerciasis [6], lymphatic filariasis [7], schistosomiasis [8], and trachoma and to administer treatment for malaria [9], [10], [11], [12]. Due to disappointing coverage with Intermittent Preventive Treatment for pregnant women (IPTp) in some situations [5], there have been attempts to deliver IPTp through a variety of community based systems [13], [14], [15]. These systems have been successful in delivering IPTp to pregnant women but there is current debate and conflicting evidence on their impact on access to other antenatal services by pregnant women.

Ensuring that the correct amount of drugs are delivered to targeted children at the most appropriate time of the year and sustaining adherence to the treatment regimen through any delivery system is a challenge. However, these challenges are not insurmountable and different ways of delivering IPTc need to be explored. In this paper we describe an evaluation of two different approaches to the delivery of IPTc in Ghana.

## Materials and Methods

The protocol of this trial and supporting CONSORT checklist are available as supporting information see Checklist S1 and Protocol S1.

### Study area

The study took place in the Jasikan district of Ghana from May to October 2006. The Jasikan District is within the semi-equatorial climatic zone and has an extended rainy season (April – November) with peaks in May-July and September-October. The district has two hospitals, 9 health centres, 17 reproductive and child health static clinics, 3 private maternity homes and 2 private clinics. The district also has 80 Expanded Programme on Immunisation (EPI) outreach clinics. In children under the age of five years resident in the study area in 2006 who were treated for clinical malaria the day28 PCR corrected adequate clinical and parasitological cure rate was 75% (95% CI 69,80) for SP (n = 241) and 85% (95% CI 69,80) for AS+AQ (n = 241) (Kweku M et al in preparation).

### Study design and conduct

**Figure 1:**
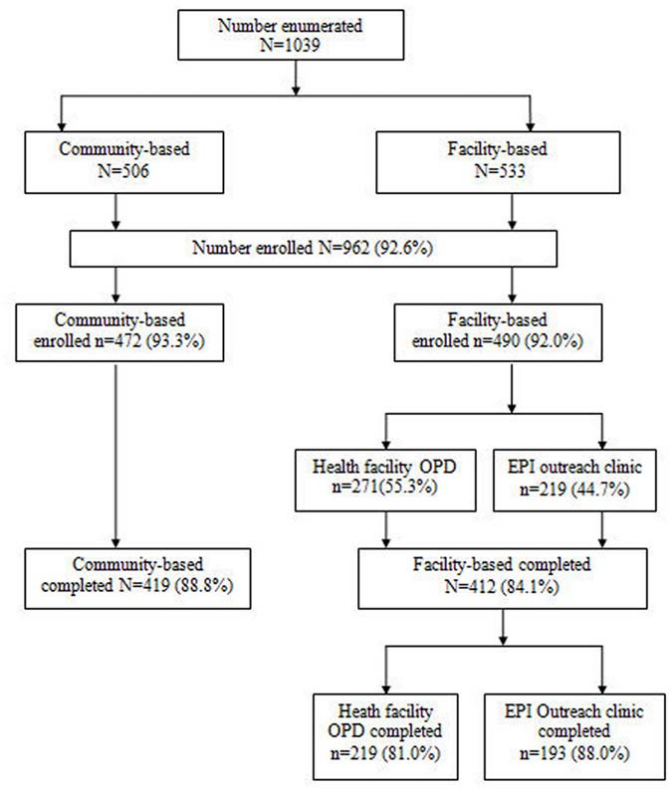
Trial Profile.

The study had two arms; IPTc was delivered either at the outpatient department of health centres and at EPI outreach clinics [health facility-based IPTc] or by community volunteers [community-based IPTc] (Figure 1). Sampling was carried out in two stages. From a sampling frame of all villages in the district with a static reproductive and child health facility (n = 17), six villages were randomly selected using a sampling interval of 3. From this sample of 6 villages, 3 villages were allocated to the facility based arm and 3 villages to the community based arm by ballot. Similarly from a sampling frame of 80 villages that had no static health facility, 6 villages were randomly selected using a sampling interval of 13. From this sample of 6 villages, 3 villages were assigned to the facility based arm (IPTc was delivered at outreach EPI clinics) and 3 to the community based arm by ballot. The study villages were on average within 5 kilometres from the static health facility. None of the health facilities were inaccessible during the study period.

Members of the district health management team and the principal investigator (MK) explained the objectives of the study to leaders of the study villages and obtained their consent for the trial. All children aged 3–59 months (1039) resident in the 12 study villages were then enumerated in May 2006. Children whose parents gave written consent (964) were enrolled into the study after a physical examination. Only three children who had a history of drug allergy were excluded. An identification card (ID card) with a unique ID number was issued to all enrolled children. The dates when IPTc was to be administered and the dose required were indicated on the ID card. Parents were advised to refer to the ID cards for information on the dates of IPTc drug administration and on whether they should go to the community-based volunteers or to the health facility to receive the study drugs. A finger prick blood sample for examination of malaria parasite was obtained at enrolment and at the end of the six month observation period.

District Health Management Team (DHMT) staff distributed drugs to the health centre staff and community-based volunteers and supervised the administration of drugs to children in May, June, September and October. In the facility-based arm, health workers who were providing outpatient treatment for sick children gave IPTc to the study children on the scheduled IPTc days and EPI nurses administered IPTc during the monthly out-reach vaccination and growth monitoring clinic. In the community based arm, children were invited to a central point in the village on the scheduled IPTc days once a month for four months and the community based volunteers administered the IPTc. Children in each arm received a three day regimen of AQ plus a single dose of SP in May, June, September and October. The first dose of the drug was administered under supervision by a health worker or by a community-based volunteer. Subsequent doses were dispensed by parents/guardians at home after training. Tablets of AQ (150 mg) and (SP) (500 mg sulfadoxine/25 mg pyrimethamine) were given as follows: ¼ tablet of each drug to 3 to 5 month-old infants, 1/2 tablet of each drug to 6 to 11 month-old infants, 3/4 of each tablet to children aged 12 to 23 months and 1 tablet of each drug to those aged 24 months or more. Tablets were crushed and mixed with water and sometimes sweetened with sugar. Drugs were administered based on age not on exact weight. Five days after administration of the first dose of IPTc, field workers visited a randomly selected 20% sample of the children in every village to ask about side effects of drugs, detect any left over medication and to enquire about the use of bednets. The number of children surveyed after each course of IPTc ranged from 73 to 91 in the community based arm and from 68 to 85 in the facility-based arm.

Community volunteers received a payment of $10 and facility-based staff a salary supplement of $10 every month for undertaking the additional work required to deliver and monitor coverage of IPTc. Further details of the economics of the two approaches to drug delivery will be presented elsewhere (Conteh et al. in preparation).

Carers were asked to take their child to the nearest health facility for examination and treatment if he or she developed any adverse event or any illness. Malaria episodes in study children were monitored through a passive surveillance system at the health centres and hospitals within the study area for a period of six months after the last dose of the first course of drug had been given (May - October 2006). A finger prick blood sample was collected from all children who presented with a history of fever or any signs suggestive of malaria before treatment with AS + AQ. Axillary temperature was measured using an electronic thermometer when a child reported at the health facility.

Blood slides were read twice at a central laboratory to give a definitive diagnosis. Laboratory assistants examined thick blood films for parasitaemia. A sample was considered negative only after 200 high power fields had been read. Parasite counts were converted to parasites per micro liter (µl), assuming a white blood cell count of 8000 leukocytes per µl of blood. If there was a discrepancy in the findings in a slide between the two initial technicians (positive or negative or a 50% or more difference in parasite density) a third, more senior microscopist read the slide and his reading was deemed to be the correct reading. A senior microscopist from the Noguchi Memorial Institute of Medical Research (NMIMR, Ghana) examined all the positive blood films and a 20% random sample of negative blood slides for quality control. Haemoglobin (Hb) concentration was measured using a Hemocue ® Photometer (Leo Diagnostics, Sweden) in children who attended study health facilities for suspected malaria or anaemia. Children with an Hb<8.0 g/dl received treatment for anaemia with iron 12.5 mg and folic acid 50 µg tablets daily for 14 days and those with an Hb = <6.0 g/dl were referred for hospital management.

### Sample size

The number of villages required for the trial was based on the following assumptions. It was assumed that coverage of IPTc in the community-based arm would be 80%, that the coefficient of variation in the IPTc coverage between villages would be 0.4 and that the number of children per village would be between 60 and 100 (average 80). On the basis of these assumptions a study with 12 clusters would have 80% power to detect a relative difference in coverage between groups of 35% at a 5% level of significance. It was also assumed that the incidence of malaria in the study area before the intervention would be 19 per 1000 child weeks and it was expected that there would be a 42% reduction in the incidence of malaria post intervention, that the coefficient of variation in the malaria incidence between villages would be 0.4 and that the number of children per village would be between 60 and 100 (average 80). On the basis of these assumptions the study had 80% power to detect a relative difference in incidence before and after the intervention of 42% at a 5% level of significance. The sample size was calculated using the formula suggested by Smith & Morrow [16].

### Analysis

The primary outcome of the trial was coverage with IPTc. Acceptable coverage was defined as the proportion of children who received at least the first dose of 3 or more courses of IPTc. Poor coverage was defined as the proportion of children who received the first dose of two or less courses of IPTc. Secondary outcomes were (1) adherence to treatment (2) the incidence of clinical malaria, (3) the incidence of anaemia, (4) the prevalence of malaria parasitaemia at the beginning and at the end of the rainy season and (5) the incidence of adverse events. Adherence to the IPTc regimen was classified as follows; highly adherent - took first supervised dose of all 4 courses; moderately adherent - took first supervised dose of three courses; poorly adherent - took the first supervised dose of two or less courses. An episode *P. falciparum* clinical malaria was defined as a history of fever during the past 72 hours or an observed temperature ≥37.5° C plus the presence of peripheral parasitaemia of any density. High parasite density malaria was defined as parasitaemia >7000 per µl in the presence of a history fever during the past 72 hours or a temperature ≥37.5°. Anaemia was defined as an Hb <8.0 g/dl.

For the analysis of coverage of IPTc, an intention to treat analysis was used in which all children who were enrolled and received the first dose of IPTc course one were included in the analysis. Statistical significance of the difference in the coverage of IPTc between the two arms was tested using logistic regression with robust standard errors to take in to account the cluster effects. The secondary outcome of adherence was defined as the proportion of children who received all three doses of each course of IPTc in the 20% random sample of children who were visited at home. Statistical significance of the differences in the adherence between the groups was tested by chi^2^test.

For the analysis of the effect of IPTc on the incidence of malaria and anaemia, we calculated person time at risk from the date of enrolment to date of the final cross sectional survey (6 months after the start of intervention). In addition, if a child received an antimalarial drug (AS+AQ) during the follow up period, we subtracted 28 day post treatment period from the person time at risk. To compare the effect of ITPc on the incidence of malaria between the two arms of the study, we used the random effects Poisson regression model and robust standard errors to allow for intra-cluster correlation and to adjust for the effect of age, and bed net use.

To compare the prevalence of malaria parasitaemia between the two arms of the study, we used a logistic regression model to adjust for the effect of clustering in the communities using robust standard errors and for the effect of age, bednet use and the number of IPTc courses administered. All statistical analysis was done using STATA version 10. (©Statacorp LP).

### Ethics

The trial was approved by the ethics committees of the London School of Hygiene & Tropical Medicine and of the Ghana Health Service/Ministry of Health (GHS/MOH).The trial was registered on the NIH clinical trials database as an amendment to the number ClinicalTrials.gov NCT00119132.

## Results

### Background characteristics

At enrolment, children in the two study groups were similar in regard to age, gender, anthropometric indices and malaria parasite prevalence (Table 1). The number of children per cluster varied but was well balanced between the two groups. It ranged from 40 to 98 children (mean 73.8) in villages where IPTc was given in the community and from 38 to108 (mean 83.2) in villages where the delivery of IPTc was facility-based.

**Table 1:** Characteristics of study children at enrolment.

Characteristics	Community-based arm	Facility-based arm
Number of clusters (N)	**6**	**6**
Number of children enumerated in each study arm n (%)	506 (48.7)	533 (51.3)
Number enrolled and received the first course of IPTc n (%)	472 (93.1)	492 (92.3)
Age (in months) (mean, SD)	26.8 (15.89)	28.5 (16.54)
Sex n (%) male	264 (55.9)	246 (50.0)
Own bednet n (%)	112 (23.7)	139 (28.3)
Own ITN n (%)	76 (16.1)	94 (19. 1)
Slept under bednet last night n (%)	73 (15.5)	107 (21.74)
Slept under ITN last night n (%)	54 (11.4)	80 (16.3)
Proportion with temperature > = 37.5°C n (%)	15 (3.2)	22 (4.5)
Malaria parasitaemia n (%)	51 (10.8)	61 (12.4)
Parasitaemia density>7000/µl n (%)	13 (2.8)	15 (3.1)
Underweight n (%)	118 (25.0)	123 (25.0)
Wasted n (%)	80 (16.9)	76 (15.5)
Stunted n (%)	35 (7.4)	49 (10.0)
BMI(mean, SE)	0.158 (0.026)	0.157 (0.025)

* Information obtained by interviewing parents/guardians of participants (use of insecticide treated net or untreated net was distinguished).

There was no statistically significant difference in the proportion of households who owned an insecticide treated net (ITN) between villages in the community based arm and the facility based arm (16.1 vs 19.2%; p = 0.281). Similarly, there was no statistically significant difference in the proportion of children who slept under an ITN on the night before the survey between villages in the two arms (11.4% vs 16.3% p = 0.196).

### Coverage with IPTc and adherence

The proportion of children who received 3 or more courses of IPTc ranged from 72.9% to 94.8% in the community-based arm and from 58.5% to 94.9% in the facility-based arm villages. The mean coverage of IPTc was slightly higher in the community based arm than in the facility-based arm (90.5 vs 86.6%; OR 1.47, 95% CI 0.63, 3.45) (Table 2). The proportion of children who received all four courses of IPTc was also slightly higher in the community based arm than in the facility-based arm (69.1 vs 65.5%; OR = 1.18; 95% CI 0.62, 2.22). However these differences in coverage of IPTc between the two arms were not statistically significant.

**Table 2:** Comparison of coverage of IPTc between the two arms.

Variables	Facility based arm	Community based arm
Number of clusters	6	6
Total number of children	492	472
Number of children who received at least the first dose of ≥3 course of IPTc	426 (86.6)$	427 (90.5)$
Odds ratio* of receiving at least the first dose of ≥3 course of IPTc	1.00	1.47 (0.63, 3.45) £

* Clustering adjusted for using logistic regression with robust standard errors.

$ Percentages; p = 0.06.

£ 95% confidence limits.

Among the 650 children who were assessed for adherence to IPTc doses, adherence to 3 doses of any course of IPTc ranged from 85% to 100% and there was no difference in the adherence to IPTc between the community based arm and the facility based arm (Table 3).

**Table 3:** Adherence to Intermittent Preventive Treatment Doses.

Outcome	Course 1 Community-based N = 91 n (%)	Course 1 Facility-based N = 85 n (%)	Course 2 Community-based N = 90 n (%)	Course 2 Facility-based N = 84 n (%)	Course 3 Community-based N = 73 n (%)	Course 3 Facility-based N = 68 n (%)	Course 4 Community-based N = 80 n (%)	Course 4 Facility-based N = 79 n (%
Received three doses	84 (92.3)	75 (88.2)	80 (88.9)	73 (86.9)	62 (84.9)	65 (95.6)	80 (100.0)	77 (97.5)
Received two doses	4 (4.4)	8 (9.4)	7 (7.8)	9 (10.7)	4 (5.5)	3 (4.4)	-	-
Received one dose or none	3 (3.3)	2 (2.4)	3 (3.3)	2 (2.4)	7 (9.6)	0 (0.0)	-	2 (2.5)

Forty-four percent (110/248) parents/guardians interviewed reported that they had used the ID card to determine the IPTc drug administration day and where they should take their child to receive treatment. Those who did not use the ID card gave the following reasons: forgot 80 (32.3%), could not read 41(16.5%) and did not understand what was written on the card 17 (6.9%).

After the administration of each course of IPTc, drug distributors were asked to record on the ID card that the child had received the first dose of drug under supervision. Eighty-one percent (783/962) of ID cards were available to assess the record of IPTc administration. Recording of IPTc drug administration on ID cards was significantly higher for children who received IPTc from community-based volunteers than for children who received their IPTc from facility-based health workers (65% vs 39% respectively) OR = 2.90 (95% CI 1.57, 5.39 p<0.001).

### Adverse events reported during the intervention period

No serious adverse event attributable to use of the study drugs was recorded. One death occurred during the observation period. This child became unwell before the fourth course of drug administration. The probable cause of death ascertained by verbal autopsy was a brain abscess.

Some children spat or vomited drugs immediately after their administration due to the bitter taste of amodiaquine. In the villages where IPTc distribution was community based, 3.5% (23/650) of the children required a repeat dose of IPTc because of vomiting compared to 2.5% (16/650) of children resident in villages where IPTc distribution was facility-based.

One hundred and fifty-three of the 650 children (23.5%) visited at home five days after administration of the first dose of IPTc experienced at least one adverse event (Table 4). Common adverse events noted by parents/guardians were vomiting/spitting drugs (10.9%), drowsiness/general bodily weakness (8.0%). Other adverse events noted were diarrhoea, body itch, abdominal pains, common cold and a swollen face. The reported incidence of adverse events fell with time and by the fourth course of drug administration, less than 2% of children reported any adverse event.

**Table 4:** Reported symptoms during the five days post IPTc administration.

Symptom	Course 1 N = 177 n (%)	Course 2 N = 173 n (%)	Course 3 N = 141 n (%)	Course 4 N = 159 n (%)	Total N = 650 n (%)
Drowsiness/weakness	20 (11.3)	24 (13.9)	5 (3.5)	3 (1.9)	52 (8.0)
Vomited study drugs	19 (10.7)	26 (15.0)	17 (12.1)	9 (5.7)	71 (10.9)
Vomiting later but not drug	2 (1.1)	1 (0.6)	3 (2.1)	0	6 (0.9)
Diarrhoea	2 (1.1)	2(1.2)	1 (0.7)	0	5 (0.8)
Restless/body itch	5 (2.8)	1 (0.6)	0	0	6 (0.9)
Abdominal pains	1 (0.6)	0	0	0	1 (0.2)
Common cold	3 (1.7)	0	0	0	3 (0.5)
Swollen face	3 (1.7)	0	3 (2.1)	0	6 (0.9)
Convulsion	0	0	1 (0.7)	0	1 (0.2)
Palpitation	1 (0.6)	0	1 (0.7)	0	2 (0.3)
Yellowish urine	1 (0.6)	0	0	0	1 (0.2)
Any adverse event	38 (21.5)	31 (17.9)	9 (6.4)	3 (1.9)	153 (23.5)

### Incidence and prevalence of malaria and anaemia

The incidences of clinical malaria, microscopically confirmed malaria of any parasite density and high parasite density were lower in children in the facility based arm than in the community based arm (Table 5). There was no statically significant difference in the prevalence of parasitaemia between the two arms at the pre implementation survey (10.2% vs 11.5%; OR 1.14, 95% CI 0.45, 2.87; p = 0.78) or at the post implementation survey (7.5% vs 6.7%; OR 0.88, CI 0.26, 2.96; p = 0.84).

**Table 5:** Comparison of incidence of malaria between the groups.

Outcome	Community based arm (n = 6)			Facility based arm (n = 6)			Rate Ratio*	P-value
	Events	PYAR	Incidence/1000PYAR	Events	PYAR	Incidence/1000PYAR		
Treated for malaria	53	133.0	398.5	13	136.5	95.2	0.24 (0.06, 0.99)	0.005
Confirmed malaria (any parasite density)	18	133.0	135.3	2	136.5	14.6	0.15 (0.02, 0.73)	0.022
Confirmed malaria (parasite density >7000/µl)	13	133.0	97.7	1	136.5	7.3	0.08 (0.01, 0.86)	0.037

PYAR person years at risk.

* Rate ratio-adjusted for clustering effect, age, ITN use and gender.

## Discussion

This study has shown that the coverage achieved for four courses of IPTc was slightly higher employing a community-based rather than a health facility-based delivery system. Both systems achieved more than 60% coverage for all four courses and over 80% coverage for 3 or more courses. However, this required good supervision and support from a District Health Management Team and the study team.

In Ghana there are many communities without a static health facility and, during the rainy season which is also the farming season, approximately 25% of the population is not regularly reached by EPI outreach clinics. Thus, although a facility-based delivery system had a relatively high coverage (86.6%) in this study, a substantial proportion of children would not have access to if IPTc is delivered exclusively through the facility-based approach.

It is likely that children who would not have access to IPTc through the health facility-based delivery system would be those living in the most inaccessible and deprived areas of the district and would be those most at risk from malaria. Delivering IPTc through the routine health system will require extra effort to reach the deprived population and adequate supervision to ensure that records are kept properly. In this area of Ghana where the study was done, a combination of health facility-based and community-based approach might be needed to maximise the impact of IPTc. In other areas, different approaches may be appropriate to achieve maximum coverage.

Although no serious adverse event attributable to the administration of IPTc with AQ + SP was observed, a significant proportion of caretakers (24%) reported mild adverse events within five days of administration of IPTc. However, in the absence of a placebo group it cannot be determined how many of these reported symptoms were due to the drug and how many to associated minor illnesses. Nevertheless, this is a concern if IPTc is to be rolled out on a national scale. The main adverse events observed in this study were bodily weakness and drowsiness and vomiting of drugs (18.9%). The reported incidence of adverse events fell over time after educating caretakers to feed children with food containing sugar at the time of drug administration. Appropriate health education on adverse events and actions to mitigate them should be a priority for any national IPTc programmes.

Most children (66/75) who reported at the health facility with a history of fever received treatment for malaria but only 27% (20/75) of the children had malaria parasitaemia. This high use of antimalarial drugs is partly due to the current treatment guidelines in Ghana that all children <5 years of age who report at the health facility with fever should be treated for malaria unless there is a clear alternative cause for the fever.

The reasons for the lower incidence of malaria in the facility based arm than in the community based arm are not clear. We speculate that background incidence of malaria was higher in the rural villages of the community based arm than the villages with a static health facility. However, both arms of the study had rural and semi-rural villages and the prevalence of fever and malaria parasitaemia was not different between the groups at the baseline.

We conclude that seasonal IPTc using community-based or facility-based delivery system can achieve high coverage in children aged 3 to 59 months. However, a facility based approach alone would not reach children living in areas without access to formal health care system. Adverse events such as drowsiness, general bodily weakness and vomiting of drugs may be concern for using SP +AQ for IPTc. However, IPTc using four courses of SP+AQ in areas with a prolonged seasonal transmission probably can reduce the burden of malaria.

## Supporting Information

Checklist S1 CONSORT Checklist
(0.05 MB DOC)

Protocol S1 Trial Protocol
(0.61 MB DOC)
