# Supplementary material for: Options for the Delivery of Intermittent Preventive Treatment for Malaria to Children: A Community Randomised Trial
Source: PLoS One. 2009 Sep 30;4(9):e7256. doi: 10.1371/journal.pone.0007256 (PMC2748713; doi:10.1371/journal.pone.0007256)
Supplement: Protocol S1 — Trial Protocol (0.61 MB DOC) [file pone.0007256.s002.doc]

**TITLE OF PROPOSAL:** Comparison of two approaches to delivering intermittent preventive treatment for malaria in children: a pilot study in Ghana

**PRINCIPAL INVESTIGATOR(S):**

Margaret Kweku London School of Hygiene and Tropical Medicine (LSHTM) & Ghana Health Service (GHS), Hohoe

**COLLABORATORS AND AFFILIATIONS:**

Jayne Webster, Daniel Chandramohan, Brian Greenwood LSHTM

Martin Adjuik Navrongo Research Centre

Fred Binka INDEPTH Network.

Samuel Abudey Ghana Health Services

**PROPOSED STARTING DATE:** First of April, 2006

**DURATION:** 12 months

**1. SUMMARY**

Intermittent preventive treatment in children (IPTc) is a promising new approach to malaria control. This approach involves giving anti-malarial drugs to 3-59 month old children at defined periods annually. Studies in Senegal and Mali show that IPTc can reduce the burden of malaria in children substantially in the Sahel and sub-Sahel regions of West Africa. It is unclear which will be the most appropriate delivery system for IPTc under routine programme conditions in order to achieve both equitable and sustainable coverage, and to maximise adherence to IPTc regimes. We propose a pilot study on ways in which IPTc can be scaled-up in routine programme conditions. The coverage of IPTc (four treatment courses of amodiaquine+ sulphadoxine-pyrimethamine during the rainy season) achieved by a network of community based volunteers will be compared to that achieved by routine Health centre and EPI/growth monitoring staff in a randomised trial. Five health centres and their catchment population will be randomly allocated to one these two approaches. The outcomes of the study will be measured by pre- and post intervention cross sectional surveys.

**2.0 BACKGROUND TO THE STUDY**

2.1 **Introduction**

It is estimated that in 2002, 400 million episodes of clinical malaria occurred in sub-Saharan Africa (Snow et al et al, 2005) and 94% of global deaths attributable to malaria occurred in this region (Bryce et al. 2005). One promising approach is the administration of intermittent preventive treatment for malaria (IPT) to infants (IPTi) or to children under five years old (IPTc), which involves administration of a predefined number of treatment courses of antimalarial drugs at specified time intervals. Administration of sulfadoxine/pyrimethamine (SP) to infants at the time of the second and third DPT and measles vaccinations reduced the incidence of malaria by 59% in an area with perennial transmission in Tanzania (schellenberg et al. 2001). However, in an area with highly seasonal transmission of malaria in Ghana SP-IPTi reduced the incidence of malaria by only 25% (Chandramohan et al. 2005). This marked variation in the protective efficacy of IPTi in Tanzania and Ghana is primarily due to the differences in the transmission dynamics of malaria between West and East Africa. In highly seasonal transmission areas of West Africa targeting IPT during the high transmission season is more appropriate than administering IPT throughout the year. Furthermore the IPTi strategy cannot be the solution for large-scale malaria control in many parts of Africa where the burden of the disease is not predominantly in infants. There is now growing evidence that season IPTc can reduce the burden of malaria substantially in Sahelian West Africa. Three courses of SP and artesunate during the high transmission season to <5 year old children reduced the incidence of malaria by 86% in Senegal (Cisse et al in press) and 40% in Mali (Dicko et al.2004).

Although there is a strong evidence base for introducing an IPTc programme, there are concerns about sustainable approaches to delivering IPTc. Community volunteers based delivery systems have been used successfully in onchocerciasis (Katabarwa et al. 2001), filariasis (Ramaiah et al. 2001), schistosomiasis (Katabarwa et al. 2005), and trachoma mass drug delivery programmes. Another option is to deliver IPTc during routine EPI/growth monitoring clinic visits. The coverage of EPI vaccination is low in many countries of West Africa in comparison to those of East and Southern Africa (Webster et al. 2005); however, in Ghana approximately 70% of 1 year olds are fully protected by all EPI vaccines. Growth monitoring visits in Ghana are higher amongst one year old children than those over one year, which may be due to the link with EPI vaccination under one year of age. It is possible to encourage caretakers to attend growth monitoring clinics through targeting health education and changing their perception of the values of these child welfare clinics. However, offering IPTc during growth monitoring visits might have an incremental positive effect and encourage caretakers to use these clinics more regularly. Conversely, it is also plausible that the increased workload of the health services staff due to this integrated delivery of IPTc and growth monitoring may lead to programme fatigue.

Ensuring that the correct amount of drugs are delivered to the target children at the most appropriate time of the year, and sustaining adherence to the treatment regimes through any delivery systems is a challenge. However, these challenges are not insurmountable and different ways of delivering IPTc need to be explored. Thus we propose to evaluate two different approaches to delivering IPTc.

**3.0 STUDY OBJECTIVES**

- 1. **Primary objective**

To compare the coverage of IPTc achieved through two different delivery systems.

- 1. **Secondary Objective**

To compare the level of adherence to the IPTc regime in the two delivery systems

To assess the impact of IPTc on the incidence of malaria

**4.0 INTERVENTIONS**

(1) Health facility based IPTc: Community mobilisation and education on IPTc offered at the EPI/growth monitoring clinic will be carried out. The IPTc drugs will be delivered at all (both static and out reach) EPI/growth monitoring clinics.

(2) Community based IPTc: A network of community volunteers (Mother co-ordinators) will be identified and trained. The IPTc drugs will be delivered every month to these volunteers by the district EPI nurse and the volunteers deliver the drug to the children in their respective villages.

**5.0 TRIAL END-POINT**

**5.1 Primary end –point**

1. The proportion of children receiving the full course of IPTc (four courses) during the rainy season.

**5.2 Secondary end-points:**

1. Proportion of IPTc courses completed per protocol (adherence to treatment regime)
2. Incidence of clinical malaria
3. Prevalence of malaria parasitaemia at the end of the rainy season.

## 6. STUDY SITE AND POPULATION

**6.1 Study Area**

The study will be carried out in the Jasikan district of Ghana. This is in the northern part of the Volta Region. It is bounded by the Togo-Ghana mountain ranges to the east, Kpando and Hohoe Districts to the south, Kadjebi District to the north and the Volta Lake to the west. The population of Jasikan district in 2004 was estimated to be 122,265. Most of the residents are subsistence farmers who grow rice and maize. The District falls within the semi-equatorial climatic zone and has an extended rainy season (May – November) with peaks in June-July and September- October. Malaria transmission peaks from May to October. *P. falciparum* is the dominant species, being responsible for all severe diseases and over 98% of clinical attacks. A few cases of clinical malaria are caused by *P.* *malariae*. In rural areas, children experience 3-6 clinical attacks of malaria a year. Over 70% of clinical attacks occur during the rainy season. Severe malaria is the most common cause of admission and death in children under five years (Jasikan district annual report 2004). Volta Region has relatively good coverage with EPI vaccines; over 80% of one year olds received the full range of vaccines according to the 2003 DHS.

**6.2 Health facility in the study area**

There are eight health centres and two hospitals in the district. Each health centre has a resident nurse or Medical Assistant. Jasikan district hospital owned by Ghana Health Service (GHS) and Worawora hospital owned by mission serve as referral hospitals. Worawora hospital has a 120 in-patient bed facilities while Jasikan district hospital has 40. Each hospital has a resident doctor.

**7.0 STUDY DESIGN AND METHODOLOGY**

**7.1 Trial Design**:

An open label trial with two arms: (1) Health facility based IPTc; (2) community based IPTc. Children in both arms will receive four courses of AQ+SP (a three day regime) in May, June, September and October (Figure 1). The EPI nurses will collect drugs from the District Health Directorate (DHD) when collecting vaccines and family planning devices for their outreach activities. The nurses will transport and deliver the drugs to the health centre staff and community-based IPTc coordinators in their catchment areas for distribution. They will also carry out health education activities about IPTc during child welfare clinics in the communities. The schedule for drug administration and number of doses per child in a year will be designed in the child’s road to health card. EPI nurses will pass on information through mothers/guardians who attend child welfare clinics or through their routine contact persons in the communities on scheduled days for drug distribution.

**Figure 1. Structure of IPTc delivery systems**

Health centre

Nurse in charge

EPI nurse

DHMT

1. Distinct Director of Health services

2. IPTc coordinator (Disease control office/Public health nurse

Mother coordinators

Deliver IPTc in the villages

Health centre and EPI/Growth monitoring staff

Deliver IPTc at EPI/growth monitoring clinics at the health centre

**7.2 Screening for eligibility**

Children whose parents or guardians wanted their wards to participate will be assessed by the research team for eligibility for enrolment into the study: Children with the following conditions will be excluded from the study: (1) severe malnutrition; (2) chronic diarrhoea; (3) history of convulsions; (4) prostration, extreme weakness (inability to stand or sit); (5) history of hypersensitivity to AQ or SP. The study staff will examine the children for these criteria at the time of pre intervention cross sectional survey and refer children having any of these conditions to the PI for the final decision.

**7.3 Obtaining informed consent**

This process will be carried out by trained field supervisors and fieldworkers using the local language Ewe, Buem and Akan in the district. All parents and care takers who will consent to participate will append their signature or right thumb print on two copies of the consent form in the presence of a witness (non-study related person) who also will sign or affix his/her thumbprint. The field supervisor/worker will also sign to indicate that (s)he has adequately informed the participant about the study and answered all questions.

**7.4 Sample size**

The sample size calculation for the primary end-point takes into account that the village and not the individual, is the unit of randomization. The number of villages required for the trial depends on the expected compliances in the village based and health centre based delivery systems, the variability in compliance between villages and the number of participating children in each village. Assuming 80% compliance in the community based IPTc delivery system and 45% or worse compliance in health centre based delivery system with a coefficient of variation between villages of 0.25, assuming that there are 60-100 children in each village, 5 villages or cluster of villages in each arm will be needed for the study. Such a study will have 80% power at 5% level of significance to show a difference in compliance.

All children eligible and their parents consented to participate in the study will be recruited. The recruited children (600-1000) will be included in the cross-sectional surveys at the beginning and end of the intervention.

**7. 5 Enumeration, recruitment and consent**

Five villages with an average population of 80 children aged less than five years will be allocated to receive IPTc distributed by health staff from five health centres. Five villages of the same size will be selected to receive IPTc from a community-based IPTc coordinator.

Once villages have been chosen in April 2006, several meetings with all potential partners (health authorities, community leaders and care givers) will be held. Meetings will be held with the district health directorate staff and assembly members from the district administration. We will then hold meetings with the chiefs and elders, opinion leaders and parents or guardians of prospective study subjects in the villages selected. Explanations will be provided to opinion leaders and parents in local languages, emphasising the potential public health impact of the study for malaria control. They will be provided with information sheets in English or in the appropriate local language (Ewe or Akan).

Enumeration of all children aged between 3-59months of age in the study area will be carried out in April 2006 and a database established before the start of the study.

Approximately, 960 children will be eligible for enrolment in the study. Enrolled subjects will be provided with an identification card on which their names, age, sex, weight at enrolment, drug code mother/guardian’s name and study number will be written to facilitate identification at each contact.

**7.6 Training of drug distributors**

The community-based IPTc coordinators will be selected in consultation with the villagers and they will receive training on how and when to administer IPTc drugs to children and how to complete the tally sheets, adverse event forms and keep records of all deaths in the study children during the period of intervention. The training will also highlight the importance of their role in the study and how it relates to that of others. At the training, they will be encouraged to do their best to ensure high quality work. The health centre distributors will be identified by the District Health Management Team and they will also receive training as above.

**7.7 Cross-sectional surveys**

Two cross-sectional surveys will be undertaken in May and November 2006. The first survey will be the pre-intervention survey which will provide baseline information about the study children and the second one will be a post- intervention survey. In both surveys, information to be collected will include the background information about the child and mother/guardian, history of allergy to any of the study drugs, bednet ownership and use, measurement of temperature and anthropometric indices. This will also involve collection of finger prick blood samples for determination of asexual and sexual parasitaemia.

**7.8, Drug administration and Follow-up:**

Drug administration to the study children will start in June 2006. The IPTc coordinators will distribute the trial medication at a central point in the village, while the health staff will do the distribution at a convenient place in the health centre. Two consecutive days will be allowed for drug distribution within the week in which drugs are received. A convenient day to start will be chosen by the parents of the participants. The first dose will be given under direct supervision of the distributors and the subsequent doses for day 2 and 3 will be given to the mothers/guardians to administer at home.

Adherence in the facility based IPTc arm will be assessed on a rolling basis throughout the study by assessing the number of courses received and correct doses of medication administered. Four days after the first dose was administered field workers will visit every village and health facility to collect drug distribution forms. They will also visit some randomly computer generated selected mothers at home to check for left over medication and ask for any side effects after the first dose of drug administration.

**7.9 Surveillance during the period of intervention**

A passive surveillance system to monitor malaria episodes in study children will be set up in the health centres within the catchment areas of the IPTc communities and in the district hospital.

Parents and guardians will be advised to come directly to a health centre if their child developed any symptoms or signs of illness. If the child has fever or any features suggestive of malaria, a finger prick blood sample will be collected for malaria parasite before treatment will be given. One slide will be prepared for each child and the slides will transported to a central laboratory for double reading and for definitive diagnosis. Those who test positive for malaria will be treated with oral quinine 10mg/kg 8hourly for 7days and will be followed on days 3 and 7 for outcome. Health centres with laboratory services, will collect blood sample for Hb. Children with Hb<8.0g/dl will receive treatment for anaemia. Every participating child who presents to the health facility for medication will be asked to produce an ID card. The information on the ID card will be compared with the master list held by the surveillance staff. Treatment at the health facility for malaria and anaemia will be free for all participating children.

Records will be kept of all admission and deaths of study children at the district hospital. A physician or a medical assistant and nurses on the ward will be asked to look out for any child with severe malaria, and any admission attributed to malaria will be carefully documented.

A surveillance supervisor will visit the health facilities fortnightly from June to October to supply logistics and collect completed record forms and prepared slides. At the same time s/he will visit IPTc coordinators to collect completed forms on community deaths. All deaths will be investigated using post-mortem questionnaire techniques and cause of death established wherever possible. The ID card will be used to confirm the identity of the deceased child and may provide some important information about the health and nutritional status of the child prior to death.

**7.10 IPTc dose regimens**

Treatment will be given during the months of June-July and September- October as follows: (AQ+SP), AQ (10mg/kg) daily for three days. SP will be given as a single dose [SP tablets containing 500mg sulfadoxine/25mm pyrimethamine will be given at a dosage of sulfadoxine (25 mg/kg) + pyrimethamine (1.25 mg/kg]. Tablets will be crushed, sweetened with sugar and mixed with water and administer orally to the children. The AQ+ SP regime was chosen because a recent study in Senegal showed that AQ+SP had the highest protective efficacy (Badara C. et al. Unpublished)

**7.11 Assessment of adverse Events**

***Eliciting and documenting adverse experiences***

All adverse events or reactions, which occur during the trial, will be documented. The nature of each experience, date and time (where appropriate) of onset, duration, severity and relationship to treatment will be established whenever possible.

***Serious Adverse Events***

A serious adverse experience is any event which is fatal, life threatening, disabling or incapacitating or results in hospitalization, prolongs a hospital stay or is associated with congenital abnormality, cancer or overdose (either accidental or intentional). Any serious adverse event that occurs within 24 hours of administration of chemoprophylaxis will be reported to the Local Safety Monitor, Chairman DSMB and director of GMP

***Data Safety Monitoring Board (DSMB)***

An independent committee will be appointed to oversee ethical and safety aspects of the study conduct. The main role of DSMB will be to review and analyse clinical safety data collected during the trial and to assess unexpected or serious adverse events (SAEs)

**7.12. Local safety monitor**

An experienced clinician will be appointed as the local safety monitor (LSM). The overall role of the LSM will be to support the clinical investigators and to act as a link between the investigators and the DSMB. All SAEs will be reported to him/her in his/her capacity as a liaison to the DSMB.

**7.13. Data Management and analysis**

All baseline, surveillance and laboratory data will be collected on forms designed for the trial.

Field and laboratory staff will be trained to follow procedures set out in the Standard Operating Procedures (SOPs.) Key data will be double entered using EPI info software and inconsistencies checked; range checks will be used for standard variables. Primary analysis will be compliance with IPTc between the five villages in the health centre group and those in the community group.

**6.0 Time Table of activities**

| **Activities** | **Duration** | **Start** | Months staring from January 2006 | | | | | | | | | | | | |
| --- | --- | --- | --- | --- | --- | --- | --- | --- | --- | --- | --- | --- | --- | --- | --- |
|  |  |  | 1 | 2 | 3 | 4 | 5 | 6 | **7** | **8** | 9 | 10 | 11 | 12 | 13 |
| High malaria transmission season | 6 months | May 06 |  |  |  |  | X | X | **X** | **X** | X | X |  |  |  |
| Development of proposal | 1 month | Jan. 06 | X |  |  |  |  |  |  |  |  |  |  |  |  |
| Submission to Ghana & LSHTM Ethics Committees | 3 months | Feb 06 |  | X |  |  |  |  |  |  |  |  |  |  |  |
| SOP/Field work (RCT) | 1month | Mar 06 |  |  | X |  |  |  |  |  |  |  |  |  |  |
| Order drugs | 2 months | Mar 06 |  |  | X |  |  |  |  |  |  |  |  |  |  |
| Recruitment and consent | 1month | April 06 |  |  |  | X |  |  |  |  |  |  |  |  |  |
| Training and sensitization | 2months | April 06 |  |  |  | X |  |  |  |  |  |  |  |  |  |
| Cross-sectional survey1 | 1 month | May 06 |  |  |  |  | X |  |  |  |  |  |  |  |  |
| Drug distribution | 4 months | June 06 |  |  |  |  | X | X |  |  | X | X |  |  |  |
| Passive surveillance | 6 months | June 06 |  |  |  |  | X | X | X | X | X | X | X |  |  |
| Data management | 6 months | June 06 |  |  |  |  | X | X | X | X | X | X | X | X |  |
| Cross-sectional survey2 | 1 month | Nov. 06 |  |  |  |  |  |  |  |  |  |  | X |  |  |
| Analysis and report writing | 6 months | Jan. 07 |  |  |  |  |  |  |  |  |  |  |  |  | X |

- 1. **Timetable and milestone for achieving objective**

###### *February 2006*

Submission to Ghana & LSHTM Ethics Committees

**March- April 2006**

Drugs and laboratory supplies procured

###### *April 2006*

Recruitment of study subjects completed.

**May 2006**

Pre- intervention **prevalence of malaria measured**

**November 006**

Post intervention prevalence of malaria and coverage of IPTc measured.

**January-Feb 2007**

Results of the study disseminated

**7.0 References**

1. Bryce J and the WHO child health epidemiology reference group. WHO estimates of the causes of death in children. Lancet, 2005;365:1147-1152
2. [Chandramohan D](http://www.ncbi.nlm.nih.gov/entrez/query.fcgi?db=PubMed&cmd=Search&itool=PubMed_Citation&term="Chandramohan+D"%5BAuthor%5D), [Owusu-Agyei S](http://www.ncbi.nlm.nih.gov/entrez/query.fcgi?db=PubMed&cmd=Search&itool=PubMed_Citation&term="Owusu-Agyei+S"%5BAuthor%5D), [Carneiro I](http://www.ncbi.nlm.nih.gov/entrez/query.fcgi?db=PubMed&cmd=Search&itool=PubMed_Citation&term="Carneiro+I"%5BAuthor%5D), [Awine T](http://www.ncbi.nlm.nih.gov/entrez/query.fcgi?db=PubMed&cmd=Search&itool=PubMed_Citation&term="Awine+T"%5BAuthor%5D), [Amponsa-Achiano K](http://www.ncbi.nlm.nih.gov/entrez/query.fcgi?db=PubMed&cmd=Search&itool=PubMed_Citation&term="Amponsa-Achiano+K"%5BAuthor%5D), [Mensah N](http://www.ncbi.nlm.nih.gov/entrez/query.fcgi?db=PubMed&cmd=Search&itool=PubMed_Citation&term="Mensah+N"%5BAuthor%5D), [Jaffar S](http://www.ncbi.nlm.nih.gov/entrez/query.fcgi?db=PubMed&cmd=Search&itool=PubMed_Citation&term="Jaffar+S"%5BAuthor%5D), [Baiden R](http://www.ncbi.nlm.nih.gov/entrez/query.fcgi?db=PubMed&cmd=Search&itool=PubMed_Citation&term="Baiden+R"%5BAuthor%5D), [Hodgson A](http://www.ncbi.nlm.nih.gov/entrez/query.fcgi?db=PubMed&cmd=Search&itool=PubMed_Citation&term="Hodgson+A"%5BAuthor%5D), [Binka F](http://www.ncbi.nlm.nih.gov/entrez/query.fcgi?db=PubMed&cmd=Search&itool=PubMed_Citation&term="Binka+F"%5BAuthor%5D), [Greenwood B](http://www.ncbi.nlm.nih.gov/entrez/query.fcgi?db=PubMed&cmd=Search&itool=PubMed_Citation&term="Greenwood+B"%5BAuthor%5D) Cluster randomised trial of intermittent preventive treatment for malaria in infants in area of high, seasonal transmission in Ghana. [BMJ.](javascript:AL_get(this, 'jour', 'BMJ.');) 2005 Oct 1; 331(7519):727-33.
3. Cisse B, Cheikh S. Sokhna, Neal Alexander, Jean Francois Trape, Brian Greewood. A double blind, randomised placebo-conrolled trial to measure the potential of sIPT with artesunate plus SP to reduce the malaria burden in Niakhar, rural Senegal Absract ASTMH 53rd Annual meeting. 2004.
4. Desai MR, Mei JV, Kariuki SK, et al., randomized controlled trial of daily iron supplementation and intermittent sulfadoxxine-pyrimethamine for the treatment of mild childhood aneamia in western Kanya. J Infect Dis.2003; 187:658-66.
5. Dicko A, Issaka Sagara, Mahamadou S, Sissoko, Ousmane Guindo, Abdou Baki I, Mamady Kone, Mahamadou A. Thera, Massambou Sacko, Ogobara K Doumbo.. Impact of intermittent preventive treatment with sulphadoxine-pyrimethamine targeting the transmission season in the incidence of clinical malaria in children of 6 months to 10 years in Kambila Mali. Absract ASTMH 53rd Annual meeting 2004.
6. Greenwood BM, David PH, Otoo-Forbes L, Allen SJ, Alonso PL, Armstrong-Schellenberg JR, Byass P, Hurwitz M, Menon A, Snow RW. Mortality and morbidity from malaria after stopping chemoprophylaxis. Trans R Soc Trop Med Hyg. 1995; 89: 629-633.
7. Greenwood BM, Greenwood AM, Bradley AK, Snow RW, Byass P, Hayes RJ, N'Jie ABH. Comparison of two strategies for control of malaria within primary health care programme in The Gambia. Lancet i: 1988; 1121-1127.
8. Katabarwa MN, Habomugisha P, Richards Jr FO, Hopkins D. Efficient and effective integration of health care delivery and development activities in rural disadvantaged communities of Uganda. [Trop Med Int Health.](javascript:AL_get(this, 'jour', 'Trop Med Int Health.');) 2005; 10: 312.
9. Massaga JJ, Kitua AY, Lemnge MM, Akida JA, Malle LN, Rønn AM, Theander TG, Bygbjerg IC. Effect of intermittent treatment with amodiaquine on anaemia and malarial fevers in infants in Tanzania: a randomized placebo-controlled trial. Lancet. 2003; 361(9372):1853-1860.
10. McGregor IA, Gilles HM, Walter JH, Davies AH, Pearson FA, Effects of heavy and repeated malaria infections on Gambian infants and children. Effects of erythrocytic parasitization. BMJ ii. 1956; 686-692.
11. Menendez C, Kahigwa E, Hirt R, Vounatsou P, Aponte JJ, Font F, Acosta CJ, Schellwnberg DM, Galindo CM, Kimario J, Urassa, Brabin B, Smith TA, Kitua AY, Tanner M, Alonso PL. Randomised placebo controlled trial of iron supplementation and malaria chemoprophylaxis for prevention of severe anaemia and malaria in Tanzanian infants. Lancet. 1997; 350:844-850.
12. Prissen Geerlings PD, Brabin BJ, Eggelte TA. Analysis of the effect of malaria chemoprophylaxis in children on haematological response, morbidity and mortality. Bull World Health Organ. 2003; 81: 206-216.
13. [Ramaiah KD](http://www.ncbi.nlm.nih.gov/entrez/query.fcgi?db=pubmed&cmd=Search&itool=pubmed_Abstract&term="Ramaiah+KD"%5BAuthor%5D), [Vijay Kumar KN](http://www.ncbi.nlm.nih.gov/entrez/query.fcgi?db=pubmed&cmd=Search&itool=pubmed_Abstract&term="Vijay+Kumar+KN"%5BAuthor%5D), [Chandrakala AV](http://www.ncbi.nlm.nih.gov/entrez/query.fcgi?db=pubmed&cmd=Search&itool=pubmed_Abstract&term="Chandrakala+AV"%5BAuthor%5D), [Augustin DJ](http://www.ncbi.nlm.nih.gov/entrez/query.fcgi?db=pubmed&cmd=Search&itool=pubmed_Abstract&term="Augustin+DJ"%5BAuthor%5D), [Appavoo NC](http://www.ncbi.nlm.nih.gov/entrez/query.fcgi?db=pubmed&cmd=Search&itool=pubmed_Abstract&term="Appavoo+NC"%5BAuthor%5D), [Das PK](http://www.ncbi.nlm.nih.gov/entrez/query.fcgi?db=pubmed&cmd=Search&itool=pubmed_Abstract&term="Das+PK"%5BAuthor%5D).Effectiveness of community and health services-organized drug delivery strategies for elimination of lymphatic filariasis in rural areas of Tamil Nadu, India.[Trop Med Int Health.](javascript:AL_get(this, 'jour', 'Trop Med Int Health.');) 2001 Dec;6(12):1062-9.
14. Schellenberg D, Menendez C, Kashigwa E, Aponte JJ, Vidal J, Tanner M , Mshinda H, Alonso P. Intermittent treatment for malaria and anaemia control at time of routine vaccination in Tanzanian infants; a randomised, placebo-controlled trial. Lancet. 2001; 357: 1471-1477.
15. Snow RW, Guerra CA, Noor AM, Myint HY, Hay SI. The global distribution of clinical episodes of Plasmodium falciparum malaria. Nature. 2005 Sep 8; 437(7030):214-7.
16. Webster J, Lines J, Bruce J, Armstrong Schellenberg JR, Hanson K. Which delivery systems reach the poor? A review of equity coverage of ever-treated nets, never-treated nets, and immunisation to reduce child mortality in Africa. Lancet Infect dis. 2005; 5(11):709-17.

**CONTRIBUTION TO THE OBJECTIVES OF THE GATES PROGRAMME**

**Indicate how this proposal meets the overall objectives of the Gates programme including capacity development in a malaria endemic country (maximum 100 words)**

**Training and Capacity Development:**

Amodiaquine plus artesunate combination is the leading option for Ghana for treatment of malaria. There is evidence that AQ+AS is an alternative to SP for IPTc. Obtaining evidence of efficacy of AQ+SP will provide an alternative to AQ+AS for IPTc before implementation as policy

**ETHICAL ISSUES**

**Discuss ethical aspects of the proposal.**

The protocol will be submitted to the London School of Hygiene & Tropical Medicine, and Ghana health service Ethics Committees for approval prior to the start of the trial.

Only those children whose parents give informed consent will be enrolled.

The study involves fingerpick blood samples from febrile children as part of routine patient care.

In addition, two finger prick samples will be obtained from all children in the trial during the cross-sectional surveys at the beginning and end of the intervention.

.

The study investigates an intervention appropriate to health needs of Ghana.

The drugs used for the trial are safe and have been used in Ghana.

Entry into the trial will not affect the health care of the participants.

Trial participants will receive free medical treatment during the period of intervention.

**Submission to LSHTM ethics committee Yes**

##### Will be submitted to LSHTM ethics committee prior to the start of the study.

**Submission to other ethics committee(s) (specify) Yes**

Will be submitted to Ghana government ethic committee prior to the start of the study.

**BUDGET (in £ sterling)**

|  | Year 1 |  |
| --- | --- | --- |
| **Personnel Cost** | ( in £) | Total (£) |
| Medical officer | 480 | 480 |
| Medical Assistants | 210 | 210 |
| Laboratory assistants | 600 | 600 |
| Laboratory technicians | 300 | 300 |
| Health facility surveillance supervisor | 150 | 150 |
| Community-based IPTc coordinators supervisors | 300 | 300 |
| Surveillance nurses | 480 | 480 |
| Drug distributors at health facility | 420 | 420 |
| Community IPTc Coordinators | 420 | 420 |
| Data Entry clerks | 480 | 480 |
| Rider | 60 | 60 |
| Driver | 60 | 60 |
| Data manager | 240 | 240 |
| Financial manager | 240 | 240 |
| **Sub Total** | **4440** | **4440** |
|  |  |  |
| **Patient care cost** | 3000 | 3000 |
| **Sub Total** | **3000** | **3000** |
| **Transport** |  |  |
| Fuel for Pick up | 800 | 800 |
| Fuel for motorbikes | 240 | 240 |
| Vehicle maintenance | 2000 | 2000 |
| Travel and Transport for EPI staff for drug distribution | 140 | 140 |
| Local travels | 1000 | 1000 |
| International travel | 2000 | 2000 |
| Communication | 500 | 500 |
| **Sub Total** | **6680** | **6680** |
| **Supplies** |  |  |
| Laboratory reagents, slides, slide boxes, lancets, and consumables | 1500 | 1500 |
| Cost of drugs for IPTc | 4000 | 4000 |
| Stationery | 500 | 500 |
| Desk top computer | 700 | 700 |
| Computing consumables | 800 | 800 |
| Procurement of microscope | 1000 | 1000 |
| Maintenance of microscopes and procurement of microscope objectives (X100), electronic thermometers, timers | 200 | 200 |
| **Sub Total** | **8700** | **8700** |
| **Cross-sectional survey** |  |  |
| Reading cross-sectional survey slides | 480 | 480 |
| Recruitment cross-sectional survey staff | 800 | 800 |
| **Sub Total** | **1280** | **1280** |
| **Other expenses** |  |  |
| Training cost for drug distribution | 120 | 120 |
| Training cost of surveillance staff and supervisors | 120 | 120 |
| Training cost of EPI staff | 36 | 36 |
| Training cost of research team members | 60 | 60 |
| Training cost for cross-sectional survey staff | 132 | 132 |
| Testing of study drugs | 150 | 150 |
| Reading passive surveillance slides | 175 | 175 |
| **Sub Total** | **793** | **793** |
| **Grand Total** | **24,893** | **24,893** |

**BUDGET JUSTIFICATION**

**Equipment**

The resources requested are for the proper conduct of the study in the Jasikan district of Ghana for one year. A desktop computer and is required for data entry. Two microscopes is required to provide laboratory support to the study.

**Personnel**

A medical officer is needed to help the PI with the study. A data manager is needed to ensure proper management of the data collected during the study. An accountant and a logistics and supplies officer are needed for proper accounting and management of logistics and funds. Two data entry clerks are needed for double entry of the data. One driver is needed to help with transportation of staff for community mobilization, conducting of surveys and monitoring of study subjects during of the study. A laboratory technician will be required for quality control. 10 community-based IPTc coordinators will be recruited for six months. Twelve field workers are needed to help with enumeration, sensitization and recruitment of study subjects and the cross-sectional surveys.

Five EPI nurses, 10 health centre staff, 10 surveillance nurses a disease control officer and a public health nurse from the DHMT will be required for the implementation of the study.

Four laboratory assistants from Hohoe DHMTs will provide laboratory support for the study

# CV OF PI AND MAIN COLLABORATORS

# CV OF PI AND MAIN COLLABORATORS

| 1. Surname   Kweku | Forename(s)  Margaret | | | Age d.o.b.  09/01/1963 | |
| --- | --- | --- | --- | --- | --- |
| 2. Degree, etc.  Medicine  Public health | | Degree  MD  MPH | University  Kharkov state Medical institute.  University of Ghana, Legon. | | Date  1991  1998 |
| 3. Posts held (with dates: please identify tenure and source of funding of present post.  District Director of Health Services. 1996-2004.  Gates Malaria Partnership (GMP) student since October 2004.  Tenure- LSHTM.  Source of funding- GMP. | | | | | |
| 4. Recent publications; also papers in press.  Seasonal prevalence of anaemia in children aged less than five years in the Hohoe district. Kweku M, Koram K, Binka F.  Monitoring and evaluation of the pilot ITN voucher scheme project in the Volta Region of Ghana., Webster Jayne, Jo lines, Kweku M.  Ability for communities to pay for artemisinin-based combination therapy. Koram K, Kweku M. | | | | | |

# CV OF FIRST MAIN COLLABORATORS

| 1. Surname   Dr. Chandramohan | | Forename(s)  Daniel | | Age | |
| --- | --- | --- | --- | --- | --- |
| Medicine | Degree  PhD | | University  LSHTM | | Date |
| 3. Posts held (with dates: please identify tenure and source of funding of present post.  Senior lecturer, Epidemiology.  Extensive clinical experience (many years) and clinical trials experience (including running ITPi trials) in Africa, and research on malaria in Tanzania. | | | | | |
| 4. Recent publications; also papers in press.  Roper C, Pearce R, Bredenkamp B, Gumede J, Drakeley C, Mosha F, Chandramohan D, Sharp B.Antifolate antimalarial resistance in southeast Africa: a population-based analysis. Lancet. 2003 Apr 5;361(9364):1174-81  Pearce RJ, Drakeley C, Chandramohan D, Mosha F, Roper C.Molecular determination of point mutation haplotypes in the dihydrofolate reductase and dihydropteroate synthase of Plasmodium falciparum in three districts of northern Tanzania. Antimicrob Agents Chemother. 2003 Apr;47(4):1347-54.  Webster J, Chandramohan D, Freeman T, Greenwood B, Kamawal AU, Rahim F, Rowland M. A health facility based case-control study of effectiveness of insecticide treated nets: potential for selection bias due to pre-treatment with chloroquine. Trop Med Int Health. 2003 Mar;8(3):196-201. Owusu-Agyei S, Fryauff DJ, Chandramohan D, Koram KA, Binka FN, Nkrumah FK, Utz GC, Hoffman SL.Characteristics of severe anemia and its association with malaria in young children of the Kassena-Nankana District of northern Ghana. Am J Trop Med Hyg. 2002 Oct;67(4):371-7. Chandramohan D, Jaffar S, Greenwood B. Use of clinical algorithms for diagnosing malaria. Trop Med Int Health. 2002 Jan;7(1):45-52.  Chandramohan D, Carneiro I, Kavishwar A, Brugha R, Desai V, Greenwood B. A clinical algorithm for the diagnosis of malaria: results of an evaluation in an area of low endemicity. Trop Med Int Health. 2001 Jul; 6(7):505-10. | | | | | |

**CV OF SECOND MAIN COLLABORATORS**

| 1. Surname   Professor Greenwood | Forename(s)  Brian |  |
| --- | --- | --- |
| 2. University: LSHTM | | |
| 3. Posts held (with dates: please identify tenure and source of funding of present post.  Manson Professor of Clinical Tropical Medicine.  Director, Malaria Centre, Gates Malaria Partnership. | | |
| 4. Recent publications; also papers in press.  Greenwood B. The use of anti-malarial drugs to prevent malaria in the population of malaria-endemic areas. America Journal of Tropical medicine and Hygiene, 2004; 70 (1):1-7  Reyburn H, Mbatia R, Drakeley C, Carneiro I, Mwakasungula E, Mwerinde O, Saganda K, Shao J, Kitua A, Olomi R, Greenwood BM, Whitty CJ.Overdiagnosis of malaria in patients with severe febrile illness in Tanzania: a prospective study. BMJ. 2004 Nov 20;329(7476):1212.  von Seidlein L, Walraven G, Milligan PJ, Alexander N, Manneh F, Deen JL, Coleman R, Jawara M, Lindsay SW, Drakeley C, De Martin S, Olliaro P, Bennett S, Schim van der Loeff M, Okunoye K, Targett GA, McAdam KP, Doherty JF, Greenwood BM, Pinder M. The effect of mass administration of sulfadoxine-pyrimethamine combined with artesunate on malaria incidence: a double-blind, community-randomized, placebo-controlled trial in The Gambia. Trans R Soc Trop Med Hyg. 2003 Mar-Apr;97(2):217-25. von Seidlein L, Greenwood BM.  Mass administrations of antimalarial drugs. Trends Parasitol. 2003 Oct;19 (10):452-60. Alloueche A, Milligan P, Conway DJ, Pinder M, Bojang K, Doherty T, Tornieporth N, Cohen J, Greenwood BM. Protective efficacy of the RTS,S/AS02 Plasmodium falciparum malaria vaccine is not strain specific. Am J Trop Med Hyg. 2003 Jan; 68(1):97-101.  Sutherland CJ, Alloueche A, Curtis J, Drakeley CJ, Ord R, Duraisingh M, Greenwood BM, Pinder M, Warhurst D, Targett GA.Gambian children successfully treated with chloroquine can harbour and transmit Plasmodium falciparum gametocytes carrying resistance genes. Am J Trop Med Hyg. 2002 Dec; 67(6):578-85. | | |

**Appendix 1**

**Jasikan district intermittent preventive treatment in children (IPTc) delivery systems study**

**INFORMATION SHEET AND CONSENT FORM**

In Ghana and in other parts of Africa, malaria and anaemia are important health problems in young children. Studies undertaken recently in Senegal and Mali have shown that these conditions can be partially prevented by giving children treatment with anti-malarial drugs on several occasions during the high transmission season, whether or not they are sick with malaria at that time. This way of trying to prevent some of the bad effects of malaria is called intermittent preventive treatment in children (IPTc).

We are not clear which will be the most appropriate delivery system for IPTc under routine programme conditions in order to achieve both equitable and sustainable coverage, and to maximise adherence to IPTc regimen. We propose a pilot study on ways in which IPTc can be scaled-up in routine programme conditions.

We need to find a safe and effective way of delivering the drugs to the children in our communities. The drugs that we are using for IPTc are sulphadoxine-pyrimethamine, usually called SP and amodiaquine AQ usually called camoquine. We have selected this drug combination because a study in Mali has shown that this combination provided a higher protection of children.

The drugs will be kept at the health centre or with the coordinator you have chosen. You will be informed at the child welfare clinic or through a contact person in your community when the drugs arrive. You will take the child together with the identification card (ID card) to the distributor and collect drugs and administer the first dose in the presence of the distributor. You will then go home to continue with the second dose the following day and third dose on the third day.

Four days after you start drug administration we will visit you at home to find out the child’s condition after taking the drug.

We want to compare the coverage of IPTc (four treatment courses of amodiaquine + sulphadoxine-pyrimethamine) during the rainy season through community-based distributors with that achieved by routine health centre and EPI/growth monitoring staff. We have selected six communities with health centres and six without health centres including your community for these two approaches and we would like you to help us to do this by enrolling your child in a study which will be conducted by staff from the District health Directorate (DHD), district hospital and health centres within Jasikan district and the London School of Tropical Medicine. This trial, which has the approval of the authorities in Ghana, is being conducted with support from the Director Initiative Fund (DIF).

If you agree that your child can take part in the trial, this is what will happen.

1. We will register your child and give him/her an ID card;
2. We will visit the community every month during the rainy season from May to November this year and he/she will be given four courses of treatment with the anti-malarial tablets described above in May and June and in September and October. We have chosen these months because the hospital data shows that children report with malaria most during these months;
3. We will follow the progress of your child until s/he for six months. Whenever your child is sick please bring him/her to the nearest health centre or hospital and show the staff the ID card. If your child is found to have malaria s/he will be treated with the recommended drug by Government for treating malaria free of charge;
4. In order to determine if the anti-malarial drugs are protecting your child against malaria and anaemia we will need to collect some small samples of blood by pricking the child’s finger on two occasions at the beginning of the study and six months later at the end of the study to confirm that the treatment has worked and any child who is found to have malaria will be treated with as mentioned above.

You are completely free to choose whether you would like your child to participate in this study or not and you are free to withdraw your child at any time. If you do not participate or withdraw your child he/she will receive all their usual vaccinations and treatment.

The information obtained on your child during the course of the study will be available only to the staff helping with the trial.

h

Do you have any questions about the study? If you want any more information at any time during the study please contact:

1. The principal investigator Dr Margaret Kweku at the district hospital, Hohoe;
2. The Doctor, Medical Assistant or the nurse of the hospital/health centre within Jasikan district;
3. The District Director of Health Services Jasikan;
4. Coordinator in your community.

If you agree to participate, please sign or thump print the consent form.

___________________________________________________________________

**CONSENT FORM**

I have understood the explanation given to me and I agree to take part in the study.

Signature: Date:

Name of parent/guardian------------------------------------------------

Signature: Date:

Name of witness------------------------------------------------

I certify that I have explained the above to -----------------------------------------------------mother/guardian of -------------------------------------------and that S/he understood what I said and has agreed to take part in the study.

Signature: Date:----------------------------

Name of field worker/supervisor--------------------------------------------

**Appendix 2**

**Jasikan IPTc delivery system**

**Screening & inclusion form for IPTc**

Sub-District ………………Community………………… date / / / /

**Child**

First Name…………………………….Last Name……………………………………

Date of birth[ ] Sex [ ] ]Weight(Kg) [ ]

**Mother/Guardian’s**

First Name………………………………Last Name…………………………………..

Age (in yrs [ ] Occupation…………………..

House No………Landmark………………………………………………………………

|  | **Inclusion criteria** | **Yes** | **No** |
| --- | --- | --- | --- |
| 1 | Age between 3 to 59 months |  |  |
| 2 | Resident in the community for at least the next six months |  |  |
| 3 | Has parent or guardian given consent |  |  |
| 4 | Child unable to take or retain medication |  |  |
| 5 | Existence of respiratory distress |  |  |
| 6 | Existence of serious or chronic illness |  |  |
|  | **Exclusion criteria** |  |  |
| 1 | History of drug allergy |  |  |
|  | **Measurement** | **Record measurement** | |
| 1 | Temperature |  |  |

**Is child eligible? [Yes] [No]**

**Enrolment status**

| Enrolled [ ] | Delivery System [ ] | Study ID number  [ ] |
| --- | --- | --- |
| Not enrolled [ ] |  | |

**Appendix 3**

**THE IDENTIFICATION CARD**

**COMPARISON OF TWO APPROACHES TO DELIVERING INTERMITTENT PREVENTIVE TREATMENT FOR MALARIA IN CHILDREN: A PILOT STUDY IN CHILDREN AGED BETWEEN 3-59 MONTHS IN THE JASIKAN DISTRICT.**

Study number (ID NO) _________

First Name ………………………Last Name…………………………………

DISTRICT**:** SUB-DISTRICT**:**

NAME OF COMMUNITY:

Study group: community-based [ ] Facility-based [ ]

DATE OF DRUG ADMINISTRATION: [1] / / [2] / / [3] / / [4] / /

**THE BEARER OF THIS CARD IS A PARTICIPANT IN THE ABOVE NAMED STUDY. TAKE FINGER PRINT BLOOD FOR HAEMOGLOBIN (HB) AND BLOOD FILM FOR MALARIA PARASITES BEFORE TREATING HIM/HER WITH THE RECOMMENDED DRUGS FOR CLINICAL MALARIA.**

**Appendix 4**

### Jasikan IPTc delivery system study

### Drug administration supervision monitoring form

Community ……………………….. Delivery system [ ]

**IPTc coordinator/ distributor:**

First name…………………………Last name……………………

**Noticed any omission** [**Y] [N]**

If yes,

| Indicator | Circle Y or N |
| --- | --- |
| Verify child’s identity by asking for the project ID card | [Y] [N] |
| Compare ID number with the master list | [Y] [N] |
| Administer correct dosage for the child’s age | [Y] [N |
| Package correct dosage for day 2 and 3 | [Y] [N] |
| Advice care giver to come back for extra dose if the child vomits drug at home |  |
| Complete drug administration form after each | [Y] [N] |
| Indicate on the ID card for the month of child took the drug | [Y] [N] |

If there omissions

Action taken by the supervisor:

……………………………………………………………………………………………………

……………………………………………………………………………………………………

……………………………………………………………………………………………………

…………………………………………………………………………………………………..

**Appendix 5**

**Jasikan IPTc Delivery System DRUG ADMINISTRATION**

|  |  | **Community: Old Ayoma** | | | | | | | | | |
| --- | --- | --- | --- | --- | --- | --- | --- | --- | --- | --- | --- |
| **Sub-district: Baika/Ayoma** | | | | | | **DATE:** | | | | | |
| **Name of IPTc coordinator/Distributor:** | | | | | | | | | | | |
| **AGE (mths)** | **DEL. SYSTEM** | | **ID NO** | **DRUG DAY 1** | **NO OF TABLETS** | | **VOMIT DAY 1 DRUG** | **IF YES, ACTION** | **OWN BEDNET** | **IF YES IS NET TREATED** | **IF YES SLEPT UNDER NET LAST NIGHT** |
| 10 | CB | | OA 001 | [ Y ] [ N ] |  | | [ Y ] [ N ] |  | [ Y ] [ N ] | [ Y ] [ N ] | [ Y ] [ N ] |
| 39 | CB | | OA 002 | [ Y ] [ N ] |  | | [ Y ] [ N ] |  | [ Y ] [ N ] | [ Y ] [ N ] | [ Y ] [ N ] |
|  |  | |  | [ Y ] [ N ] |  | | [ Y ] [ N ] |  | [ Y ] [ N ] | [ Y ] [ N ] | [ Y ] [ N ] |
|  |  | |  | [ Y ] [ N ] |  | | [ Y ] [ N ] |  | [ Y ] [ N ] | [ Y ] [ N ] | [ Y ] [ N ] |
|  |  | |  | [ Y ] [ N ] |  | | [ Y ] [ N ] |  | [ Y ] [ N ] | [ Y ] [ N ] | [ Y ] [ N ] |
|  |  | |  | [ Y ] [ N ] |  | | [ Y ] [ N ] |  | [ Y ] [ N ] | [ Y ] [ N ] | [ Y ] [ N ] |
|  |  | |  | [ Y ] [ N ] |  | | [ Y ] [ N ] |  | [ Y ] [ N ] | [ Y ] [ N ] | [ Y ] [ N ] |
|  |  | |  | [ Y ] [ N ] |  | | [ Y ] [ N ] |  | [ Y ] [ N ] | [ Y ] [ N ] | [ Y ] [ N ] |

SUMMARY

| TABLETS DISTRIBUTED | NUMBER | NO WITH BEDNETS | NO WITH ITNs |
| --- | --- | --- | --- |
| 1/4 |  |  |  |
| 1/2 |  |  |  |
| 3/4 |  |  |  |
| 1 |  |  |  |

**Appendix 6**

### Jasikan IPTc delivery system study

### Adverse event reporting form

Community ………………….

Child’s First Name………………………… Last name……………………..

Child’s ID Number: |___|___|___|___|___|

Mother’s First name……………………………Last name…..……………….

**Notified adverse events** [Y] [N]

If yes,

| Adverse event | Date onset | Time onset | End date | End time | Severity |
| --- | --- | --- | --- | --- | --- |
| General bodily weakness |  |  |  |  |  |
| drowsiness |  |  |  |  |  |
| Difficulty in breathing |  |  |  |  |  |
| Vomiting |  |  |  |  |  |
| Cannot sleep |  |  |  |  |  |
| Cannot eat |  |  |  |  |  |
| Other |  |  |  |  |  |
| Other |  |  |  |  |  |
| Other |  |  |  |  |  |

**Appendix 7**

# JASIKAN IPTc DELIVERY SYSTEM STUDY Out Patient Surveillance Form (OPS FORM)

|  | 1. ID NUMBER | | | |  | |  |  | |  |  |  | | | | | | | | | | | | | | | | | | | | | | | | |
| --- | --- | --- | --- | --- | --- | --- | --- | --- | --- | --- | --- | --- | --- | --- | --- | --- | --- | --- | --- | --- | --- | --- | --- | --- | --- | --- | --- | --- | --- | --- | --- | --- | --- | --- | --- | --- |
|  | | | | | | | | | | | |  | | | |  | | |  | | |  | | |  |  |  |  |  |  |  |  |  |  |  |  |
| 2. Name of Health facility __________________ | 3A. CLINIC CODE | | | | | | |  | |  |  |  | | |  |  |  |  |  |  |  |  | | | | | | | | | | | | | | |
|  | 3b. DELIVERY SYSTEM | | | | | | |  | |  |  |  | | |  |  |  |  |  |  |  |  | | | | | | | | | | | | | | |
| 4 .Name of child ___________________________________ | | | | | | | | | | | |  | | |  |  |  |  |  |  |  |  |  |  |  | | | | | | | | | | | |
|  | | | | | | | | | | | |  | | | | | | | | | | | | | | | | | | | | | | | | |
| 5. Date of birth (date/month/year) ………………………………………… | | | |  | |  |  |  | |  |  |  |  | | | | | | | | | | | | | | | | | | | | | | | |
|  | | | | | | | | | | | |  | | | | | | | | | | | | | | | | | | | | | | | | |
| 6. Sex of child …………………………………………….. | | **m**ale | | | **1** | |  | | **f**emale | | **2** |  | |  | | | | | | | | | | | | | | | | | | | | | | |
|  | | | | | | | | | | | |  | | | | | | | | | | | | | | | | | | | | | | | | |
| 7. Date of exam. (date/month/year) ………………………………………….. | | |  | |  | |  |  | |  |  |  |  | | | | | | | | | | | | | | | | | | | | | | | |

| Ask whether the child has any of the following symptoms | | | | | | | | | | | | | |
| --- | --- | --- | --- | --- | --- | --- | --- | --- | --- | --- | --- | --- | --- |
| 8. Can the child suckle/drink?................................................... | | | yes | **1** |  | no | | **2** |  | | dk | **8** |  |
|  | | | | | | | | | | | | | |
| 9. Fever within the past 2 days…………………………….. | | | yes | **1** |  | no | | **2** |  | | dk | **8** |  |
| 10. If the child has fever, how many days? ……………………………………………………. | | | | | | | | | | |  |  |  |
| 11. Runny nose ……………………………………………. | | | yes | 1 |  | no | 2 | |  | | dk | 8 |  |
|  | | | | | | | | | | | | | |
| 12. Cough …………………………………………………. | | | yes | 1 |  | no | 2 | |  | | dk | 8 |  |
|  | | | | | | | | | | | | | |
| 13. Difficulty in breathing ……………………………….. | | | yes | 1 |  | no | 2 | |  | | dk | 8 |  |
|  | | | | | | | | | | | | | |
| 14. Diarrhoea …………………………………………….. | | | yes | 1 |  | no | 2 | |  | | dk | 8 |  |
| 14a. If has diarrhoea, what is the type of stool | | Watery | **1** |  | Bloody | | **2** | |  | Other | | **3** |  |
|  | | | | | | | | | | | | | |
| 15.Vomiting …………………………………………. | | | yes | 1 |  | no | 2 | |  | | dk | 8 |  |
| 16. Fits during this illness ………………………… | | | yes | 1 |  | no | 2 | |  | | dk | 8 |  |
| 16a. If the child has fits, number of fits in 24 hours ……………………………… | | | | | | | | | | |  |  |  |
| 17. Itching/ scratching (pruritus)…………………… | | | yes | 1 |  | no | 2 | |  | | dk | 8 |  |
| 18. Did the child receive any of the following drugs before coming to the clinic for the present illness? | | | | | | | | | | | | | |
| A. SP (fansider) | | | yes | 1 |  | no | 2 | |  | | dk | 8 |  |
| B. Other antimalarials (specify) ______________________ | | | yes | 1 |  | no | 2 | |  | | dk | 8 |  |
| C. Antibiotics …………………………………………………… | | | yes | 1 |  | no | 2 | |  | | dk | 8 |  |
| D. Vitamins/iron …………………………………………….. | | | yes | 1 |  | no | 2 | |  | | dk | 8 |  |
| E. others (specify) ___________________________ | | | yes | 1 |  | no | 2 | |  | | dk | 8 |  |
|  | | | | | | | | | | | |  |  |
| **19. If the child had received any drug, from where?** | A. Home | | yes | 1 |  | no | 2 | |  | | dk | 8 |  |
|  | B. Shop | | yes | 1 |  | no | 2 | |  | | dk | 8 |  |
|  | C. Clinic/hosp | | yes | 1 |  | no | 2 | |  | | dk | 8 |  |
| (If others, specify) ______________________________ | D. Others | | yes | 1 |  | no | 2 | |  | | dk | 8 |  |

Please look for the following signs

| 20. Weight (kg) ………………………………………………………………….. | | | | | | | | | | | | | | | | | | | |  | |  | | · |  | Kg |
| --- | --- | --- | --- | --- | --- | --- | --- | --- | --- | --- | --- | --- | --- | --- | --- | --- | --- | --- | --- | --- | --- | --- | --- | --- | --- | --- |
|  | | | | | | | | | | | | | | | | | | | | | | | | | | |
| 21. Axillary Temp. (ºC) ………………………………………………………… | | | | | | | | | | | | | | | | | | | |  | |  | | · |  | ˚C |
|  | | | | | | | | | | | | | | | | | | | | | | | | | | |
| 22. Pale conjunctiva……. …………………………………….. | | | | | | | | | | | yes | | | | 1 | |  | | no | 2 | |  | | ns | 8 |  |
|  | | | | | | | | | | | | | | | | | | | | | | | | | | |
| 23. Jaundice ………………..………………………………….. | | | | | | | | | | | yes | | | | 1 | |  | | no | | 2 | |  | ns | 8 |  |
|  | | | | | | | | | | | | | | | | | | | | | | | | | | |
| 24. Mouth sores (stomatitis)………….………………………… | | | | | | | | | | | yes | | | | 1 | |  | | no | | 2 | |  | ns | 8 |  |
|  | | | | | | | | | | | | | | | | | | | | | | | | | | |
| 25. Pale palms………………………….………………………. | | | | | | | | | | | yes | | | | 1 | |  | | no | | 2 | |  | ns | 8 |  |
|  | | | | | | | | | | | | | | | | | | | | | | | | | | |
| 26. Skin rash (specify) _____________________________ | | | | | | | | | | | yes | | | | 1 | |  | | no | | 2 | |  | ns | 8 |  |
|  | | | | | | | | | | | | | | | | | | | | | | | | | | |
| 27. Nasal flaring ……………………………………..……….. | | | | | | | | | | | yes | | | | 1 | |  | | no | | 2 | |  | ns | 8 |  |
|  | | | | | | | | | | | | | | | | | | | | | | | | | | |
| 28.Chest indrawing ……………………………………………. | | | | | | | | | | | yes | | | | 1 | |  | | no | | 2 | |  | ns | 8 |  |
|  | | | | | | | | | | | | | | | | | | | | | | | | | | |
| 29. Respiratory rate per minute ………………………………………………………… | | | | | | | | | | | | | | | | | | | | | | |  |  |  |  |
|  | | | | | | | | | | | | | | | | | | | | | | | | | | |
| 30 Mucous membrane …………………………………… | | | | | | | | | | dry | | | | **1** | |  | | Normal | | | **2** | |  | ns | **8** |  |
|  | | | | | | | | | | | | | | | | | | | | | | | | | | |
| **31 Skin turgor ……………………………………………** | | | | | | | | | | reduced | | | | **1** | |  | | normal | | | **2** | |  | ns | **8** |  |
|  | | | | | | | | | | | | | | | | | | | | | | | | | | |
| 32. Oedema ……………………………………………......... | | | | | | | | | | | yes | | | | 1 | |  | | no | | 2 | |  | ns | 8 |  |
|  | | | | | | | | | | | | | | | | | | | | | | | | | | |
| 33. Pus in ear ………………………………………………… | | | | | | | | | | | yes | | | | 1 | |  | | no | | 2 | |  | ns | 8 |  |
|  | | | | | | | | | | | | | | | | | | | | | | | | | | |
| 34. Neck stiffness …………………………………………… | | | | | | | | | | | yes | | | | 1 | |  | | no | | 2 | |  | ns | 8 |  |
|  | | | | | | | | | | | | | | | | | | | | | | | | | | |
| 35. Level of consciousness …………………… | | | | | | | | | Normal | | **1** | |  | | Abnormal | | | | | | **2** |  | | ns | **8** |  |
|  | | | | | | | | | | | | | | | | | | | | | | | | | | |
| 36 Fontenella | | **n**ormal | **1** |  | sunken | **2** |  | bulging | | **3** |  | ns | | | **8** | |  | | not applicable | | | | | | **9** |  |
|  | | | | | | | | | | | | | | | | | | | | | | | | |  | |
|  | Take blood samples for examining malaria parasites and HB | | | | | | | | | | | | | | | | | | | | | | | | |  |
|  | | | | | | | | | | | | | | | | | | | | | | | | | | |
| 37. Blood slide taken ……………………………………….. | | | | | | | | | | | yes | | | | 1 | |  | | no | 2 | | |  | ns | 8 |  |
| **37a. If blood slide was not taken specify the reason(s) __________________________________** | | | | | | | | | | | | | | | | | | | | | | | | | | |
|  | | | | | | | | | | | | | | | | | | | | | | | | | | |
| 38. Haemoglobin checked | | | | | | | | | | | yes | | | | 1 | |  | | no | 2 | | |  | ns | 8 |  |
|  | | | | | | | | | | | | | | | | | | | | | | | | | | |
| **38a. If HB was not checked specify the reason(s) __________________________________** | | | | | | | | | | | | | | | | | | | | | | | | | | |

| 39. Blood slide result | positive | 1 |  | Negative | 2 |  | nS | 8 |  | not applicable | | | | 9 |  |
| --- | --- | --- | --- | --- | --- | --- | --- | --- | --- | --- | --- | --- | --- | --- | --- |
|  | | | | | | | | | | | | | | | |
| 40. Hb (G/DL) ………………………………………………………… | | | | | | | | | | |  |  | • |  |  |

Record your Diagnosis and the Treatment given to the child

| 41. Diagnosis _____________________________________________ | | | | | | | | | | | | | | | | |  |  |  |
| --- | --- | --- | --- | --- | --- | --- | --- | --- | --- | --- | --- | --- | --- | --- | --- | --- | --- | --- | --- |
|  | | | | | | | | | | | | | | | | | | | |
| 42. Antimalarial (specify) ____________________________ | | | | | yes | | | 1 |  | no | 2 | | |  | | | ns | 8 |  |
|  | | | | | | | | | | | | | | | | | | | |
| 43. Antibiotics (specify) ___________________________ | | | | | yes | | | 1 |  | no | | 2 | | |  | | ns | 8 |  |
|  | | | | | | | | | | | | | | | | | | | |
| 44. Antihistamine (specify)__________________________ | | | | | yes | | | 1 |  | no | | 2 | | |  | | ns | 8 |  |
|  | | | | | | | | | | | | | | | | | | | |
| 44. Other drugs (Speccify) ____________________________ | | | | | yes | | | 1 |  | no | | 2 | | |  | | ns | 8 |  |
|  | | | | | | | | | | | | | | | | | | | |
| 45. Management plan | Home care | 1 |  | admitted | | 2 |  | referred | | | | | 8 |  | | ns | | 8 |  |
|  | | | | | | | | | | | | | | | | | | | |
| 46. Outpatient surveillance form completed by………………………………………………….. | | | | | | | | | | | | | | | | |  |  |  |

**DK** = Do not know; **NS** = Not sure

**Appendix 8**

# JASIKAN IPTC DELIVERY SYSTEM Study In Patient Surveillance Form (IPS FORM)

# Part 1 to be filled in by research nurse or attending physician

|  | 1. ID NUMBER | | | |  | |  |  | |  |  |  | | | | | | | | | | | | | | | | | | | | | | | | |
| --- | --- | --- | --- | --- | --- | --- | --- | --- | --- | --- | --- | --- | --- | --- | --- | --- | --- | --- | --- | --- | --- | --- | --- | --- | --- | --- | --- | --- | --- | --- | --- | --- | --- | --- | --- | --- |
|  | | | | | | | | | | | |  | | | |  | | |  | | |  | | |  |  |  |  |  |  |  |  |  |  |  |  |
| 2.Name of Hospital __________________ | 3.DELIVERY SYSTEM | | | | | | |  | |  |  |  | | |  |  |  |  |  |  |  |  | | | | | | | | | | | | | | |
| 4 .Name of child ___________________________________ | | | | | | | | | | | |  | | |  |  |  |  |  |  |  |  |  |  |  | | | | | | | | | | | |
| 5. Date of birth (date/month/year) ……………………………… | | | |  | |  |  |  | |  |  |  |  | | | | | | | | | | | | | | | | | | | | | | | |
| 6. Sex of child …………………………………………….. | | **m**ale | | | **1** | |  | | **f**emale | | **2** |  | |  | | | | | | | | | | | | | | | | | | | | | | |
| 7. Date of Admission. (date/month/year) ………………………… | | |  | |  | |  |  | |  |  |  |  | | | | | | | | | | | | | | | | | | | | | | | |

| Ask whether the child has any of the following symptoms | | | | | | | | | | | | | |
| --- | --- | --- | --- | --- | --- | --- | --- | --- | --- | --- | --- | --- | --- |
| 8. Can the child suckle/drink?..................................... | | | yes | **1** |  | no | | **2** |  | | dk | **8** |  |
| 9. Fever within the past 2 days…………………………….. | | | yes | **1** |  | no | | **2** |  | | dk | **8** |  |
| 10. If the child has fever, how many days? ……………………………………… | | | | | | | | | | |  |  |  |
| 11. Runny nose …………………………………… | | | yes | 1 |  | no | 2 | |  | | dk | 8 |  |
| 12. Cough …………………………………………… | | | yes | 1 |  | no | 2 | |  | | dk | 8 |  |
| 13. Difficulty in breathing ………………………… | | | yes | 1 |  | no | 2 | |  | | dk | 8 |  |
| 14. Diarrhoea ………………………………………… | | | yes | 1 |  | no | 2 | |  | | dk | 8 |  |
| 14a. If has diarrhoea, what is the type of stool | | Watery | **1** |  | Bloody | | **2** | |  | Other | | **3** |  |
| 15.Vomiting ………………………………………… | | | yes | 1 |  | no | 2 | |  | | dk | 8 |  |
| 16. Fits during this illness ………………………… | | | yes | 1 |  | no | 2 | |  | | dk | 8 |  |
| 16a. If the child has fits, number of fits in 24 hours …………………………… | | | | | | | | | | |  |  |  |
| 17. Itching/ scratching (pruritus)………………… | | | yes | 1 |  | no | 2 | |  | | dk | 8 |  |
| 18. Did the child receive any of the following drugs before coming to the clinic for the present illness? | | | | | | | | | | | | | |
| A. SP ( fansider) | | | yes | 1 |  | no | 2 | |  | | dk | 8 |  |
| B. Other antimalarials (specify) ____________________ | | | yes | 1 |  | no | 2 | |  | | dk | 8 |  |
| C. Antibiotics ……………………………………………… | | | yes | 1 |  | no | 2 | |  | | dk | 8 |  |
| D. Vitamins/iron …………………………………………… | | | yes | 1 |  | no | 2 | |  | | dk | 8 |  |
| E. others (specify) ___________________________ | | | yes | 1 |  | no | 2 | |  | | dk | 8 |  |
|  | | | | | | | | | | | |  |  |
| **19. If the child had received any drug, from where?** | A. Home | | yes | 1 |  | no | 2 | |  | | dk | 8 |  |
|  | B. Shop | | yes | 1 |  | no | 2 | |  | | dk | 8 |  |
|  | C. Clinic/hosp | | yes | 1 |  | no | 2 | |  | | dk | 8 |  |
| (If others, specify) ______________________________ | D. Others | | yes | 1 |  | no | 2 | |  | | dk | 8 |  |

Please look for the following signs

| 20. Weight (kg) ………………………………………………………………….. | | | | | | | | | | | | | | | | | | | |  | |  | | · |  | Kg |
| --- | --- | --- | --- | --- | --- | --- | --- | --- | --- | --- | --- | --- | --- | --- | --- | --- | --- | --- | --- | --- | --- | --- | --- | --- | --- | --- |
|  | | | | | | | | | | | | | | | | | | | | | | | | | | |
| 21. Axillary Temp. (ºC)………………………………………………………… | | | | | | | | | | | | | | | | | | | |  | |  | | · |  | ˚C |
| 22. Pale conjunctiva ……………….……………….. | | | | | | | | | | | yes | | | | 1 | |  | | no | 2 | |  | | ns | 8 |  |
|  | | | | | | | | | | | | | | | | | | | | | | | | | | |
| 23. Jaundice ..……………………………………………….. | | | | | | | | | | | yes | | | | 1 | |  | | no | | 2 | |  | ns | 8 |  |
|  | | | | | | | | | | | | | | | | | | | | | | | | | | |
| 24. Mouth sores (stomatitis)…………………………………… | | | | | | | | | | | yes | | | | 1 | |  | | no | | 2 | |  | ns | 8 |  |
|  | | | | | | | | | | | | | | | | | | | | | | | | | | |
| 25. Pale palm ………………………………………………….. | | | | | | | | | | | yes | | | | 1 | |  | | no | | 2 | |  | ns | 8 |  |
|  | | | | | | | | | | | | | | | | | | | | | | | | | | |
| 26. Skin rash (specify) _____________________________ | | | | | | | | | | | yes | | | | 1 | |  | | no | | 2 | |  | ns | 8 |  |
|  | | | | | | | | | | | | | | | | | | | | | | | | | | |
| 27. Nasal flaring ………………………………………….. | | | | | | | | | | | yes | | | | 1 | |  | | no | | 2 | |  | ns | 8 |  |
|  | | | | | | | | | | | | | | | | | | | | | | | | | | |
| 28.Chest indrawing ………………………………………. | | | | | | | | | | | yes | | | | 1 | |  | | no | | 2 | |  | ns | 8 |  |
|  | | | | | | | | | | | | | | | | | | | | | | | | | | |
| 29. Respiratory rate per minute ………………………………………………………… | | | | | | | | | | | | | | | | | | | | | | |  |  |  |  |
|  | | | | | | | | | | | | | | | | | | | | | | | | | | |
| 30 Mucous membrane …………………………………… | | | | | | | | | | dry | | | | **1** | |  | | Normal | | | **2** | |  | ns | **8** |  |
|  | | | | | | | | | | | | | | | | | | | | | | | | | | |
| **31 Skin turgor ……………………………………………** | | | | | | | | | | reduced | | | | **1** | |  | | normal | | | **2** | |  | ns | **8** |  |
|  | | | | | | | | | | | | | | | | | | | | | | | | | | |
| 32. Oedema ……………………………………………... | | | | | | | | | | | yes | | | | 1 | |  | | no | | 2 | |  | ns | 8 |  |
|  | | | | | | | | | | | | | | | | | | | | | | | | | | |
| 33. Pus in ear …………………………………………….. | | | | | | | | | | | yes | | | | 1 | |  | | no | | 2 | |  | ns | 8 |  |
|  | | | | | | | | | | | | | | | | | | | | | | | | | | |
| 34. Neck stiffness ………………………………………. | | | | | | | | | | | yes | | | | 1 | |  | | no | | 2 | |  | ns | 8 |  |
|  | | | | | | | | | | | | | | | | | | | | | | | | | | |
| 35. Level of consciousness …………………… | | | | | | | | | Normal | | **1** | |  | | Abnormal | | | | | | **2** |  | | ns | **8** |  |
|  | | | | | | | | | | | | | | | | | | | | | | | | | | |
| 36 Fontenella | | **n**ormal | **1** |  | sunken | **2** |  | bulging | | **3** |  | ns | | | **8** | |  | | not applicable | | | | | | **9** |  |
|  | | | | | | | | | | | | | | | | | | | | | | | | |  | |
|  | Take blood samples for examining malaria parasites and HB | | | | | | | | | | | | | | | | | | | | | | | | |  |
| 37. Blood slide taken ……………………………………….. | | | | | | | | | | | yes | | | | 1 | |  | | no | 2 | | |  | ns | 8 |  |
| **37a. If blood slide was not taken specify the reason(s) __________________________________** | | | | | | | | | | | | | | | | | | | | | | | | | | |
| 38. Haemoglobin checked | | | | | | | | | | | yes | | | | 1 | |  | | no | 2 | | |  | ns | 8 |  |
|  | | | | | | | | | | | | | | | | | | | | | | | | | | |
| **38a. If HB was not checked specify the reason(s) __________________________________** | | | | | | | | | | | | | | | | | | | | | | | | | | |

| 39. Blood slide result | positive | 1 |  | Negative | 2 |  | nS | 8 |  | not applicable | | | | 9 |  |
| --- | --- | --- | --- | --- | --- | --- | --- | --- | --- | --- | --- | --- | --- | --- | --- |
|  | | | | | | | | | | | | | | | |
| 40. Hb (G/DL) ……………………………………………………………………... | | | | | | | | | | |  |  | • |  |  |
| 41. Part 1 Inpatient surveillance form completed by……………………………………………. | | | | | | | | | | | | |  |  |  |

Part 2 to be filled by attending physician

Record your Diagnosis and the Treatment given to the child

|  | | | | | | | | | | | 1. ID NUMBER | | | | | | | | | |  | | | |  | | | | |  | | | | | |  | | | | |  | |  | | | |
| --- | --- | --- | --- | --- | --- | --- | --- | --- | --- | --- | --- | --- | --- | --- | --- | --- | --- | --- | --- | --- | --- | --- | --- | --- | --- | --- | --- | --- | --- | --- | --- | --- | --- | --- | --- | --- | --- | --- | --- | --- | --- | --- | --- | --- | --- | --- |
|  | | | | | | | | | | | | | | | | | | | | | | | | | | | | | | | | | | | | | | | | | | | | |  | |
| 42.Crackles/ bronchial breathing…………………………….. | | | | | | | | | | | | yes | | | | | 1 | | |  | | | no | | | 2 | | | | |  | | | | | | ns | | | 8 | |  | | |  | |
|  | | | | | | | | | | | | | | | | | | | | | | | | | | | | | | | | | | | | | | | | | | | | |  | |
| 43.Wheezing/ ronchi…………………………………………. | | | | | | | | | | | | yes | | | | | 1 | | |  | | | no | | | | 2 | | | | |  | | | | | ns | | | 8 | |  | | |  | |
|  | | | | | | | | | | | | | | | | | | | | | | | | | | | | | | | | | | | | | | | | | | | | |  | |
| 44. Hepatomegaly……………………………………………. | | | | | | | | | | | | yes | | | | | 1 | | |  | | | no | | | | 2 | | | | |  | | | | | ns | | | 8 | |  | | |  | |
|  | | | | | | | | | | | | | | | | | | | | | | | | | | | | | | | | | | | | | | | | | | | | |  | |
| 44. Splenomegaly…………………………………………….. | | | | | | | | | | | | yes | | | | | 1 | | |  | | | no | | | | 2 | | | | |  | | | | | ns | | | 8 | |  | | |  | |
|  | | | | | | | | | | | | | | | | | | | | | | | | | | | | | | | | | | | | | | | | | | | | |  | |
| 46. Chest X-ray | | Normal | | | | 1 | |  | Abnormal | | | | 2 | |  | | Not taken | | | | | | | | | | | 3 | | |  | | | ns | | | | | | 8 | |  | | |  | |
|  | | | | | | | | | | | | | | | | | | | | | | | | | | | | | | | | | | | | | | | | | | | | |  | |
| 47. Lumbar puncture | | Normal | | | | 1 | |  | Abnormal | | | | 2 | |  | | Not done | | | | | | | | | | | 3 | | |  | | | ns | | | | | | 8 | |  | | |  | |
|  | | | | | | | | | | | | | | | | | | | | | | | | | | | | | | | | | | | | | | | | | | | | |  | |
| **Summary of treatment received during admission** | | | | | | | | | | | | | | | | | | | | | | | | | | | | | | | | | | | | | | | | | | | | |  | |
| 49. Oral SP…………………………………………………. | | | | | | | | | | | | yes | | | | | 1 | | |  | | | no | | | 2 | | | | |  | | | | | | ns | | | 8 | |  | | |  | |
|  | | | | | | | | | | | | | | | | | | | | | | | | | | | | | | | | | | | | | | | | | | | | |  | |
| 50. Oral Amodiaquine……………………………………… | | | | | | | | | | | | yes | | | | | 1 | | |  | | | no | | | | 2 | | | | |  | | | | | ns | | | 8 | |  | | |  | |
|  | | | | | | | | | | | | | | | | | | | | | | | | | | | | | | | | | | | | | | | | | | | | |  | |
| 51. Oral Quinine……………………………………………. | | | | | | | | | | | | yes | | | | | 1 | | |  | | | no | | | | 2 | | | | |  | | | | | ns | | | 8 | |  | | |  | |
|  | | | | | | | | | | | | | | | | | | | | | | | | | | | | | | | | | | | | | | | | | | | | |  | |
| 52. Parenteral Quinine……………………………………… | | | | | | | | | | | | yes | | | | | 1 | | |  | | | no | | | | 2 | | | | |  | | | | | ns | | | 8 | |  | | |  | |
|  | | | | | | | | | | | | | | | | | | | | | | | | | | | | | | | | | | | | | | | | | | | | |  | |
| 53.Other Antimalarial (specify) _________________________ | | | | | | | | | | | | yes | | | | | 1 | | |  | | | no | | | | 2 | | | | |  | | | | | ns | | | 8 | |  | | |  | |
|  | | | | | | | | | | | | | | | | | | | | | | | | | | | | | | | | | | | | | | | | | | | | |  | |
| 54. Antibiotic (specify)________________________________ | | | | | | | | | | | | yes | | | | | 1 | | |  | | | no | | | | 2 | | | | |  | | | | | ns | | | 8 | |  | | |  | |
|  | | | | | | | | | | | | | | | | | | | | | | | | | | | | | | | | | | | | | | | | | | | | |  | |
| 55.Antipyretic (Specify) ______________________________ | | | | | | | | | | | | yes | | | | | 1 | | |  | | | no | | | | 2 | | | | |  | | | | | ns | | | 8 | |  | | |  | |
|  | | | | | | | | | | | | | | | | | | | | | | | | | | | | | | | | | | | | | | | | | | | | |  | |
| 56. Anticonvuslant/sedative (specify) ____________________ | | | | | | | | | | | | yes | | | | | 1 | | |  | | | no | | | | 2 | | | | |  | | | | | ns | | | 8 | |  | | |  | |
|  | | | | | | | | | | | | | | | | | | | | | | | | | | | | | | | | | | | | | | | | | | | | |  | |
| 57. Antihistamine specify)_____________________________ | | | | | | | | | | | | yes | | | | | 1 | | |  | | | no | | | | 2 | | | | |  | | | | | ns | | | 8 | |  | | |  | |
|  | | | | | | | | | | | | | | | | | | | | | | | | | | | | | | | | | | | | | | | | | | | | |  | |
| 58. Anti-inflammatory (Specify) _______________________ | | | | | | | | | | | | yes | | | | | 1 | | |  | | | no | | | | 2 | | | | |  | | | | | ns | | | 8 | |  | | |  | |
|  | | | | | | | | | | | | | | | | | | | | | | | | | | | | | | | | | | | | | | | | | | | | |  | |
| 59. TB treatment (specify) _____________________________ | | | | | | | | | | | | yes | | | | | 1 | | |  | | | no | | | | 2 | | | | |  | | | | | ns | | | 8 | |  | | |  | |
|  | | | | | | | | | | | | | | | | | | | | | | | | | | | | | | | | | | | | | | | | | | | | |  | |
| 60. Micronutrients (vits, iron- specify)____________________ | | | | | | | | | | | | yes | | | | | 1 | | |  | | | no | | | | 2 | | | | |  | | | | | ns | | | 8 | |  | | |  | |
|  | | | | | | | | | | | | | | | | | | | | | | | | | | | | | | | | | | | | | | | | | | | | |  | |
| 61. Other drugs (Specify) ____________________________ | | | | | | | | | | | | yes | | | | | 1 | | |  | | | no | | | | 2 | | | | |  | | | | | ns | | | 8 | |  | | |  | |
|  | | | | | | | | | | | | | | | | | | | | | | | | | | | | | | | | | | | | | | | | | | | | |  | |
| 62. IV Fluids ………………………………………………… | | | | | | | | | | | | yes | | | | | 1 | | |  | | | no | | | | 2 | | | | |  | | | | | ns | | | 8 | |  | | |  | |
|  | | | | | | | | | | | | | | | | | | | | | | | | | | | | | | | | | | | | | | | | | | | | |  | |
| 63. Blood transfusion ………………………………………... | | | | | | | | | | | | yes | | | | | 1 | | |  | | | no | | | | 2 | | | | |  | | | | | ns | | | 8 | |  | | |  | |
|  | | | | | | | | | | | | | | | | | | | | | | | | | | | | | | | | | | | | | | | | | | | | |  | |
| 64. Date of discharge. (date/month/year) ………………………… | | | | | | | | | | | | | | | | | | |  | | |  | | |  | | | | |  | | | | | |  | | | | |  | |  | | | |
|  | | | | | | | | | | | | | | | | | | | | | | | | | | | | | | | | | | | | | | | | | | | | |  | |
| 65. Outcome | Alive | | 1 |  | Dead | | 2 |  | | Absconded | | | | 3 | |  | | Transferred | | | | | | | | | | | 4 | | | |  | | NS | | | | | 8 | | | |  | |  |
|  | | | | | | | | | | | | | | | | | | | | | | | | | | | | | | | | | | | | | | | | | | | | | |  |
| 66.Final Diagnosis 1 (Underlying cause)__________________________________ | | | | | | | | | | | | | | | | | | | | | | | |  | | | | | |  | | | | | | | | |  | | |  | | | |  |
|  | | | | | | | | | | | | | | | | | | | | | | | | | | | | | | | | | | | | | | | | | | | | | |  |
| 67.Final Diagnosis (Immediate cause)__________________________________ | | | | | | | | | | | | | | | | | | | | | | | |  | | | | | |  | | | | | | | | |  | | |  | | | |  |
|  | | | | | | | | | | | | | | | | | | | | | | | | | | | | | | | | | | | | | | | | | | | | | |  |
| 68. Part 2 Inpatient surveillance form completed by…………………………………………….. | | | | | | | | | | | | | | | | | | | | | | | | | | | | | | | | | | | | | |  | |  | |  | | | |  |

Total cost [¢ ]

**Appendix 9**

### Jasikan IPTc delivery system study

### Monitoring form

Community……………………………………………………..Date------/-------/2006

Child’s first name…………………………………..Last name………………………...

Date of birth -----/------/--------Child’s ID Number [ ] Delivery system [ ]

Mother/guardian’s First name…………………………Last name……………………

Age[ ] Educational level…………………………Occupation……………………..

Noticed any adverse event during drug administration[Y] [N]

If yes,

| Adverse event | Date onset | Time onset | End date | End time | Severity[[1]](#footnote-2)1 |
| --- | --- | --- | --- | --- | --- |
|  |  |  |  |  |  |
|  |  |  |  |  |  |
|  |  |  |  |  |  |

| Indicator | Circle Y or N |
| --- | --- |
| Own bednet | [Y] [N] |
| If yes since when did you get the net |  |
| If YES has net been treated within the past 12 months | [Y] [N] |
| How much did you pay for the net | **¢** |
| Did child sleep under net last night | [Y] [N |
| Look at the net | [Y] [N] |
| Brand of net (the label on the net) |  |
| Did the child complete the three doses of IPTc drugs (For 3 days?) | [Y] [N] |
| If no how many doses (days) completed | [ ] |
| Child vomited drug at home | [Y] [N] |
| If yes Action taken (collect extra dose and repeated) | [Y] [N] |

1. ^1^ Severity: 1=Mild, 2=Moderate, 3=Severe [↑](#footnote-ref-2)
